# Neural-net-based cell deconvolution from DNA methylation reveals tumor microenvironment associated with cancer prognosis

**DOI:** 10.1093/narcan/zcae022

**Published:** 2024-05-15

**Authors:** Yoshiaki Yasumizu, Masaki Hagiwara, Yuto Umezu, Hiroaki Fuji, Keiko Iwaisako, Masataka Asagiri, Shinji Uemoto, Yamami Nakamura, Sophia Thul, Azumi Ueyama, Kazunori Yokoi, Atsushi Tanemura, Yohei Nose, Takuro Saito, Hisashi Wada, Mamoru Kakuda, Masaharu Kohara, Satoshi Nojima, Eiichi Morii, Yuichiro Doki, Shimon Sakaguchi, Naganari Ohkura

**Affiliations:** Department of Experimental Immunology, Immunology Frontier Research Center, Osaka University, Suita, Osaka, Japan; Integrated Frontier Research for Medical Science Division, Institute for Open and Transdisciplinary Research Initiatives (OTRI), Osaka University, Suita, Osaka, Japan; Department of Experimental Immunology, Immunology Frontier Research Center, Osaka University, Suita, Osaka, Japan; Department of Basic Research in Tumor Immunology, Graduate School of Medicine, Osaka University, Suita, Osaka, Japan; Pharmaceutical Research Division, Shionogi & Co., Ltd., Toyonaka, Osaka, Japan; Faculty of Medicine, Osaka University, Suita, Osaka, Japan; Department of Hepato-Biliary-Pancreatic Surgery, Hyogo Medical University, Nishinomiya, Hyogo, Japan; Division of Hepato-Biliary-Pancreatic Surgery and Transplantation, Department of Surgery, Graduate School of Medicine, Kyoto University, Kyoto, Kyoto, Japan; Division of Hepato-Biliary-Pancreatic Surgery and Transplantation, Department of Surgery, Graduate School of Medicine, Kyoto University, Kyoto, Kyoto, Japan; Faculty of Life and Medical Sciences, Doshisha University, Kyotanabe, Kyoto, Japan; Department of Pharmacology, Yamaguchi University Graduate School of Medicine, Ube, Yamaguchi, Japan; Shiga University Medical Science, Otsu, Shiga, Japan; Department of Experimental Immunology, Immunology Frontier Research Center, Osaka University, Suita, Osaka, Japan; Department of Experimental Immunology, Immunology Frontier Research Center, Osaka University, Suita, Osaka, Japan; Pharmaceutical Research Division, Shionogi & Co., Ltd., Toyonaka, Osaka, Japan; Department of Clinical Research in Tumor Immunology, Graduate School of Medicine, Osaka University, Suita, Osaka, Japan; Department of Dermatology, Graduate School of Medicine, Osaka University, Suita, Osaka, Japan; Department of Dermatology, Graduate School of Medicine, Osaka University, Suita, Osaka, Japan; Department of Gastroenterological Surgery, Graduate School of Medicine, Osaka University, Suita, Osaka, Japan; Department of Gastroenterological Surgery, Graduate School of Medicine, Osaka University, Suita, Osaka, Japan; Department of Clinical Research in Tumor Immunology, Graduate School of Medicine, Osaka University, Suita, Osaka, Japan; Department of Gastroenterological Surgery, Graduate School of Medicine, Osaka University, Suita, Osaka, Japan; Department of Obstetrics and Gynecology, Graduate School of Medicine, Osaka University, Suita, Osaka, Japan; Department of Pathology, Graduate School of Medicine, Osaka University, Suita, Osaka, Japan; Department of Pathology, Graduate School of Medicine, Osaka University, Suita, Osaka, Japan; Department of Pathology, Graduate School of Medicine, Osaka University, Suita, Osaka, Japan; Department of Gastroenterological Surgery, Graduate School of Medicine, Osaka University, Suita, Osaka, Japan; Department of Experimental Immunology, Immunology Frontier Research Center, Osaka University, Suita, Osaka, Japan; Department of Experimental Immunology, Institute for Life and Medical Sciences, Kyoto University, Kyoto, Kyoto, Japan; Department of Experimental Immunology, Immunology Frontier Research Center, Osaka University, Suita, Osaka, Japan; Department of Basic Research in Tumor Immunology, Graduate School of Medicine, Osaka University, Suita, Osaka, Japan

## Abstract

DNA methylation is a pivotal epigenetic modification that defines cellular identity. While cell deconvolution utilizing this information is considered useful for clinical practice, current methods for deconvolution are limited in their accuracy and resolution. In this study, we collected DNA methylation data from 945 human samples derived from various tissues and tumor-infiltrating immune cells and trained a neural network model with them. The model, termed MEnet, predicted abundance of cell population together with the detailed immune cell status from bulk DNA methylation data, and showed consistency to those of flow cytometry and histochemistry. MEnet was superior to the existing methods in the accuracy, speed, and detectable cell diversity, and could be applicable for peripheral blood, tumors, cell-free DNA, and formalin-fixed paraffin-embedded sections. Furthermore, by applying MEnet to 72 intrahepatic cholangiocarcinoma samples, we identified immune cell profiles associated with cancer prognosis. We believe that cell deconvolution by MEnet has the potential for use in clinical settings.

## Graphical Abstract

**Graphical Abstract:**
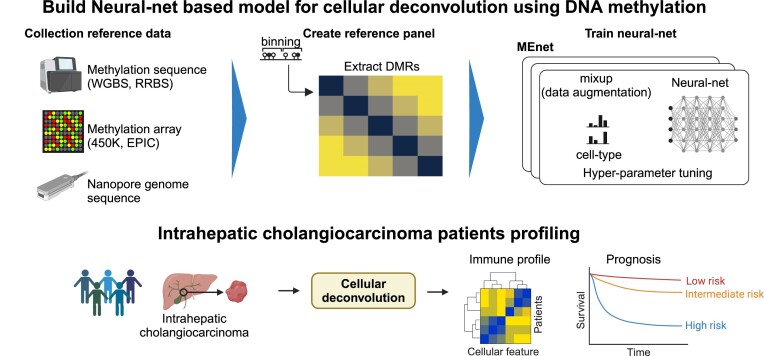

## Introduction

DNA methylation is recognized as a principal epigenetic modification that dictates cellular identity. When DNA undergoes demethylation, chromatin becomes accessible, leading to the promotion of mRNA transcription of genes in regulatory regions through histone modifications (1). While gene expression is susceptible to changes in cellular states and environmental conditions, DNA methylation consistently represents the lineage and characteristics of cells (2,3). In recent years, extensive profiling of DNA methylation across various cell types has elucidated their cellular specificity and biological significance (3). Notably, in immune cells, DNA methylation is used as a robust marker for the suppressive function of regulatory T (Treg) cells (4). Additionally, DNA methylation has been reported to be useful for identifying the primary tumor sites in cancers of unknown primary origin due to its robustness (5).

Deconvolution is a computational strategy to infer cell proportion using bulk sample data and reference cell type-specific data. Applying deconvolution to RNA expression or DNA methylation data enables the calculation of cell proportion within tissues. The significance of deconvolution techniques using DNA methylation has been increasingly evident in both clinical and research settings. For example, deconvolution was used in the analysis of tumor-infiltrating immune cells (6). Additionally, deconvolution of circulating cell-free DNA (cfDNA), which cannot be assessed by RNA-seq, aids in cancer monitoring and in the elucidation of the pathology of diseases such as COVID-19 (7), myocardial infarction (8), and neuromyelitis optica (9). Furthermore, in predicting the efficacy of immune checkpoint inhibitor treatments, profiling tumor-infiltrating immune cells has been shown to be more effective than focusing on the expression of single molecules like PD-L1 (10). These observations suggest that deconvolution is an attractive approach for analyzing clinical samples compared to flow cytometry and multiplex immunohistochemistry, which are not practical for personalized medicine in a hospital. In addition, DNA would be more suitable for clinical tests compared to RNA due to its stability and ease of handling. Notably, using nanopore sequencers, it is now feasible to directly sequence DNA without bisulfite conversion, enabling the profiling of DNA methylation within 3 h in clinics (11).

Despite the technological advancements, obstacles still remain with cell classification based on DNA methylation, particularly in accurately estimating tumor-infiltrating immune cells. Specifically, existing studies lacked a comprehensive definition for certain subsets of immune cells, including finer distinctions like activated or naïve states within T cells (3,6,12,13). Moreover, while many existing techniques have been carefully benchmarked, they mainly focused on evaluating peripheral blood mononuclear cell (PBMC) deconvolution in an epigenome-wide association study (EWAS) with assumed cellular frequency corrections (14–16). For evaluating tumors and cfDNA, it would be crucial to use a large reference including a variety of tissues and immune cells showing different cell states. Methodologically, traditional deconvolution methods often define a signature matrix for each cell type and use linear models or models with limited flexibility, such as SVR (3,6,13,17,18). However, these approaches might not be suitable for inferring multiple cell types due to the low adaptability to large datasets with high variance.

To address these issues, we developed a DNA methylation-based cell deconvolution method using neural-net architecture and named it MEnet (Methylation-based Estimation of cellular NETworks). MEnet is specifically designed to adapt flexibly to complex methylation profiles of large datasets using neural-net. Additionally, we constructed a reference consisting of a variety of cell types and immune cells with different states to overcome the limitations of specified references by integrating data from external datasets such as ENCODE (19), Roadmap (20), Blueprint (21), and the human methylome atlas (3) and original data. We trained our model using these references to adopt a wide range of cell diversity.

In this study, we also explored the potential application of MEnet to actual diseases by analyzing tumor tissues of intrahepatic cholangiocarcinoma (ICC). ICC is a malignant tumor originating from the bile ducts within the liver. It has a diverse background, with factors ranging from parasites, autoimmune diseases and organic solvents to lifestyle habits, and possesses a tumor microenvironment composed of various cell subtypes (22,23). ICC often has a poor prognosis due to its aggressive nature and resistance to treatment, necessitating the development of new therapeutic strategies and biomarkers (22). Understanding how the tumor microenvironment relates to the biology and the clinical characteristics of ICC may provide new insights into this intractable cancer. Through the analysis of tumor tissues from 72 ICC patients, we demonstrate that the prognosis of ICC patients, such as life span and recurrence time, can be estimated by the tumor microenvironment inferred by MEnet.

## Materials and methods

### Ethics

Surgically removed tumor tissues and formalin-fixed paraffin-embedded (FFPE) samples from patients with kidney, lung, colorectal, ovarian, bladder, gastric cancer, or melanoma were collected. Peripheral blood was obtained from ovarian cancer patients or healthy donors. The study using human samples was reviewed and approved by the Research Ethics Committee of Osaka University (708-10) and Kyoto University (G1023-4) and carried out in accordance with the committee's guidelines and regulations. Written informed consent was obtained from all donors.

### DNA extraction from FFPE sections

DNA was extracted from four 10 μm sections using the QIAamp DNA FFPE Tissue Kit. During this process, Deparaffinization Solution (QIAGEN) was used for deparaffinization. The obtained DNA was incubated overnight at 65°C to perform decrosslinking.

### Conventional dendritic cell populations from PBMCs

PBMCs were purified by gradient density centrifugation using Lymphoprep (Axis Shield, Dundee, UK). The cells from each fraction were stained using the following markers: CD45 (Biolegend: 304 048, Biolegend BV510 Mouse Anti-Human CD45, Clone: HI30), CD3 (Biolegend: 300 450, Biolegend PE/Dazzle Mouse Anti-Human CD3, Clone: UCHT1), CD16 (Biolegend: 302 006, Biolegend FITC Mouse Anti-Human CD16, Clone: 3G8), CD19 (Biolegend: 302 252, Biolegend PE/Dazzle Mouse Anti-Human CD19, Clone: HIB19), CD20 (Biolegend: 302 348, Biolegend PE/Dazzle Mouse Anti-Human CD20, Clone: 2H7), CD56 (Biolegend: 318 348, Biolegend PE/Dazzle Mouse Anti-Human CD56, Clone: HCD56), CD14 (Biolegend: 325 604, Biolegend FITC Mouse Anti-Human CD14, Clone: HCD14), CD15 (Biolegend: 323 004, Biolegend FITC Mouse Anti-Human CD15, Clone: W6D3), CD11c (Biolegend: 301 608, Biolegend PE-Cy7 Mouse Anti-Human CD11c, Clone: 3.9), HLA-DR (Biolegend: 307 610, Biolegend APC Mouse Anti-Human HLA-DR, Clone: L243), CD141 (Biolegend: 344 104, Biolegend PE Mouse Anti-Human CD141, Clone: M80), CD1c (Biolegend: 331 526, Biolegend BV421 Mouse Anti-Human CD1c, Clone: L161).

Dendritic cell fractions were sorted by MACSQuant Tyto (Miltenyi Biotec) and subsequently lysed in 400 μl of Lysis Buffer, which comprised 100 mM NaCl, 10 mM Tris–HCl at pH 8.0, 50 mM EDTA and 0.5% SDS. The lysates were treated with Proteinase K and incubated overnight at 55°C to ensure complete lysis. DNA was then extracted using a standard phenol–chloroform extraction method and precipitated with ethanol.

The dendritic cell fractions were sorted as follows: conventional dendritic cell type 1; lineage marker-negative (CD3, CD16, CD19, CD20, CD56), CD14^−^ CD15^−^ CD45^+^ HLA-DR^+^ CD11c^+^ CD141^+^; Conventional dendritic cell type 2; lineage marker-negative (CD3, CD16, CD19, CD20, CD56) CD14^−^ CD15^−^ CD45^+^ HLA-DR^+^ CD11c^+^ CD1c^+^.

### Tumor-infiltrating lymphocyte

The tumor slices were dissociated using GentleMACS (Miltenyi Biotec). The cells from each fraction were stained using the following markers: CD4 (Thermo: 48-0049-42, CD4 Monoclonal Antibody (RPA-T4), eFluor™ 450, eBioscience™, Clone: RPA-T4), CD8 (BD: 557 746, BD Pharmingen™ PE-Cy™7 Mouse Anti-Human CD8, Clone: RPA-T8), CD45RA (BD: 555 488, BD Pharmingen™ FITC Mouse Anti-Human CD45RA, Clone: HI100), CD25 (BD: 555 432, BD Pharmingen™ PE Mouse Anti-Human CD25, Clone: M-A251), PD1 (BioLegend: 329 924, Brilliant Violet 605™ anti-human CD279 (PD-1), Clone: EH12.2H7), CCR8 (BD: 566 898, BD Pharmingen™ APC Mouse Anti-Human CCR8, Clone: 433H), Live/Dead (Thermo: L34976, LIVE/DEAD™ Fixable Near-IR Dead Cell Stain Kit). The cell fractions were sorted by FACSAria Ⅲ (BD Biosciences) as follows: Exhausted CD8: CD3^+^ CD8^+^ CD45RA^−^ PD1^+^; Effector CD8: CD3^+^ CD8^+^ CD45RA^−^ PD1-; Effector Treg: CD3^+^ CD4^+^ CD25^high^ CCR8^+^; Treg: CD3^+^ CD4^+^ CD25^high^.

Sorted cells were dissolved in 400 μl of Lysis Buffer (homemade), and Proteinase K (Thermo Fisher Scientific) was added, followed by overnight dissolution at 55°C. DNA extraction was performed using the phenol–chloroform method, followed by the collection of the aqueous layer from chloroform, and then purified using the Geno Plus Genomic DNA Extraction MiniPrep Kit (VIOGENE).

### Tumor tissue

For skin cancer samples, DNA was extracted from fresh tumor slices using the QIAamp DNA Mini Kit (QIAGEN). For ICC samples, frozen specimens preserved in the Department of Surgery of Kyoto University as tumor/non-tumor were used as samples. Genomic DNA was isolated from each frozen tissue using PureLink^®^ Genomic DNA Kits (Invitrogen). QC (Quality Control) and Whole Genome Bisulfite Sequencing (WGBS) were conducted as described above.

### cfDNA

Serum samples were purified by gradient density centrifugation using Lymphoprep (Axis Shield, Dundee, UK). Serum samples were centrifuged at 20 000 × g for 5 min using a swing rotor to pellet any contaminating cellular debris. The clarified supernatant (1 ml) was then subjected to cfDNA extraction using the QIAamp Circulating Nucleic Acid Kit (QIAGEN) according to the manufacturer's instructions.

### Library preparation and sequence

DNA was fragmented using the S220 Focused-ultrasonicator and ME220 Focused-ultrasonicator (Covaris). The fragmented DNA was then used to prepare libraries with the NEBNext Enzymatic Methyl-seq Kit (E7120L). The completed libraries were sequenced on the DNBSEQ-G400 (MGI) platform using 100bp paired-end sequencing.

For tumor-infiltrating lymphocytes, DNA processing was outsourced to Takara Bio Inc., where DNA was fragmented using S220 Focused-ultrasonicator and ME220 Focused-ultrasonicator (Covaris), followed by library preparation with the NEBNext Enzymatic Methyl-seq kit, and sequencing was performed using the NovaSeq 6000 (Illumina). Some samples of liver tissue and peripheral blood were sequenced using Nanopore, following the protocol described in the following section.

### Nanopore sequencing

DNA sequencing was performed using a MinION sequencer (Oxford Nanopore Technologies). The library was prepared using the Rapid Sequencing Kit (SQK-RAD004) following the manufacturer's protocol. R9.4.1 (FLO-MIN106D) flow cells were used for sequencing, which was carried out for 72 h. The raw sequencing data were processed using MinKNOW (version 23.04.3). This procedure was in accordance with the official guidelines provided by Oxford Nanopore Technologies.

### Methylation data preparations

For publicly available methylation quantification data, we directly used the methylation rates provided. For datasets without pre-calculated methylation rates, we determined methylation rates using the following procedures. For WGBS data, Reads were trimmed using trim_galore (0.6.7). Alignment was performed using Bismark (v0.23.1) (24). PCR duplicates were removed using duplicate_bismark. Then, Methylation rates were extracted using bismark_methylation_extractor. For RRBS data, the WGBS protocol was followed, but with the addition of the –rrbs option in trim_galore, the duplicate_bismark step was skipped. For Nanopore direct DNA sequencing, base calling was performed using guppy_basecaller (v5.0.16 + b9fcd7b) with the configuration -c dna_r9.4.1_450bps_modbases_dam-dcm-cpg_hac_prom.cfg. Methylation status identification and methylation rate extraction were conducted using fast5mod (v1.0.4). The reference genome used was GRCh38.p13. Detailed procedures are available at https://github.com/yyoshiaki/MEnet/tree/main/create_ref.

For the deconvolution input, to ensure consistency across platforms and to achieve robustness against noise, we performed genome-wide tiling using Bedtools map at 1000 bp intervals. We then calculated the average methylation rate and the total number of methylated and unmethylated CpG reads for downstream analysis. For methylation arrays, we calculated the average methylation rate of the loci contained in each bin and subsequently computed hypothetical methylated and unmethylated read counts to achieve a total read count of 5.

### Model comparison

From the projects of Roadmap, ENCODE, AMED-CREST and BLUEPRINT, we collected data from 151 samples spanning 20 categories, ensuring at least five samples per category. We performed genome-wide tiling at 1000 bp intervals and used the average methylation rate of each bin for model comparison. We extracted variably methylated bins for input using the following strategy: For each bin, we conducted a chi-squared test on the total methylated versus unmethylated read counts for each minor group category against all other categories. Referring to the previous study (25), we sought to identify variable regions. Specifically, to weigh the reliability based on read numbers, we conducted chi-squared tests for each bin. Bins were selected as characteristic regions if they had a false discovery rate within the top 500 and a methylation rate difference of >60% between the category and all other categories, resulting in 25 486 feature regions. Using the average methylation rate of the extracted regions, we conducted training and testing at the Major group level. For training, we used only samples that included a minimum of three samples, preparing a total of 138 samples. For testing, we used data from cohorts not used in training. Categories with more than 15 samples were limited to 15 samples, resulting in a total of 96 test samples. Missing values were imputed using the median methylation rate of the same bin from the training data. We created simulation data for mixed data by randomly selecting 5 samples from the test samples and summing them, producing 200 simulated samples.

For the Multi-Layer Perceptron (MLP), we used two intermediate layers with 1000 nodes each, applied a dropout of 0.1 to the intermediate layers, and implemented batch normalization for each layer. We used Stochastic Gradient Descent (SGD) with a learning rate of 0.005. Without mixup, we ran for 500 epochs, and with mixup, we ran for 10 000 epochs. We prepared comparison models including LightGBM (with parameters ‘learning_rate’: 0.5, ‘objective’: ‘mae’, ‘metric’: ‘mae’, ‘num_leaves’: 9, ‘bagging_fraction’: 0.7, ‘feature_fraction’: 0.7), Random Forest (using default parameters), Support Vector Regression (using default parameters), and NNLS (where we averaged the methylation rate by category for training samples to create a reference). Comparisons with existing methods were conducted using the same reference as NNLS. ARIC (v0.1) (17) was used with default parameters. CIBERSORT (26) was run using an R script downloaded from https://cibersortx.stanford.edu/runcibersortx.php. We employed EpiDISH (2.12.0) (16) to infer RPC (16), CP/QP (27) and CIBERSORT. Accuracy was measured using the mean squared error calculated with scikit-learn. Time measurements were conducted using an Intel Xeon Platinum 8180 @ 2.500 GHz and NVIDIA Tesla V100 PCIe 32 GB.

### Feature region extractions for MEnet inputs

To ensure the inclusion of regions vital to both Major and Minor Groups, we employed a two-step extraction process. Initially, we calculated the read count for each bin and selected those bins that fell within the 0.5–99.5 percentile range. In the first step, to identify characteristic regions at the Major Group level, we aggregated the methylated and unmethylated read counts for each Major Group category and conducted a chi-squared test between that category and all other categories. In the second step, for each Major Group containing multiple Minor Groups, we conducted tests to pinpoint characteristic regions for each Minor Group. Specifically, we aggregated the methylated and unmethylated read counts for each Minor Group and performed a chi-squared test between that Minor Group and other Minor Groups within the same Major Group. Lastly, bins that exhibited a methylation rate difference of >40% between the category and all others, and had *P*-values within the smallest 2000 were selected as feature regions.

For the visualization of the extracted feature regions, we conducted median imputation and then visualized the data using a heatmap. For UMAP-based visualization, after scaling the imputed matrix, we applied PCA and used PC1–PC200 to compute UMAP using umap-learn (v0.5.2). The enrichment analysis of the feature regions was conducted using the GenomicDistributions package in R.

### Architecture of MEnet

The main architecture of MEnet is an MLP model built with a Python package PyTorch (v1.6.0). Cross-validation (CV) was conducted using StratifiedShuffleSplit in scikit-learn v0.24.1, where we performed stratified splits for each of the 6-fold based on labels. For handling missing values, we employed median imputation. For hyperparameter tuning, we utilized the CmaEsSampler from Optuna (v2.6.0), selecting from the following search space based on the validation loss calculated by OneHotCrossEntropy: number of layers: [2,10], hidden layers: [30,3000], dropout rate: [0,0.3], activation functions: {relu, tanh, leakyrelu}, optimizers: {Adam, RMSprop, SGD}, and learning rate: [1e-5, 1e-1]. We set the maximum number of epochs to 200 000 and implemented EarlyStopping (patience = 1000). For training data augmentation, we randomly selected up to 15 samples, applied random weights, and introduced missing values with a dropout rate of 0.2. These values were then imputed based on the median methylation rate of the training data. The training was conducted using MEnet train on an Intel Xeon Platinum 8180 @ 2.500GHz and NVIDIA Tesla V100 PCIe 32GB. We implemented distributed learning using MySQL.

### Subsampling

Sequence data (Supplementary Table S1, S2) were downloaded from fastq, while fastq files consisting of multiple runs were merged. For subsampling reads, seqkit sample (v2.1.0) was used to extract each proportion of reads. In this case, we used five different seeded reads. For the calculation of methylation rate, trim_galore (RRBS with the –rrbs option), bismark, deduplicate_bismark (WGBS only), and bismark_methylation_extractor were used with default parameters, and the reference sequence Gencode v34. For the simulation of mixed samples, we integrated the output files of bismark created by the subsampling step randomly and used the weighted average of sum_len calculated by seqkit stats as the target value. MEnet, ARIC, CIBERSORT, RPC and CP/QP was used for the prediction on the generated simulation set, and scikit-learn was used to calculate the prediction accuracy.

### Calculation of tumor purity and immune cell scores using MEnet

We applied MEnet to the Illumina 450k DNA methylation datasets from various cancer types in The Cancer Genome Atlas (TCGA). For each cancer type, we used the corresponding tissue-specific DNA methylation data to estimate the cell-type fractions in the bulk tumor samples. To calculate the tumor purity score, we summed the estimated fractions of all cell types except for blood cells, fibroblasts, adipocytes, endothelial cells, cardiovascular cells, and muscle cells, and then subtracted this sum from 1. This approach assumes that the aforementioned cell types primarily constitute the tumor stroma, while the remaining cell types represent the tumor cells. For the immune cell score, we simply summed the estimated fractions of all immune cell types present in the reference matrix. We then benchmarked the MEnet-derived tumor purity scores against various established methods, including the gene expression-based ESTIMATE algorithm (28), the copy number variation-based ABSOLUTE method (29), immunohistochemistry (IHC), and a consensus approach combining all three methods (consensus purity estimate, CPE) (30). Similarly, we compared the MEnet-derived immune cell scores with the gene expression-based immune score from the DNA methylation-based leukocyte unmethylation for purity (LUMP) score (30).

### Human tumor dissociation

Tumor samples were minced and enzymatically dissociated with the Tumor Dissociation Kit, human (Miltenyi Biotec), according to the manufacturer's instructions protocol. Cell suspensions were filtered through a 70 μm cell strainer and isolated by Percoll (Sigma-Aldrich) gradient centrifugation.

### Flow cytometry analysis

For flow cytometry analyses, cells underwent staining procedures. Initially, dead cells were identified and marked using dead cell staining. Subsequently, cells were stained with fluorophore-conjugated antibodies CD45 (Biolegend: 304 048, Biolegend BV785 Mouse Anti-Human CD45, Clone: HI30), CD3 (Biolegend: 300 424, Biolegend AF700 Mouse Anti-Human CD3, Clone: UCHT1), CD4 (Biolegend: 317 440, Biolegend BV711 Mouse Anti-Human CD4, Clone: OKT4), CD8 (Biolegend: 301 048, Biolegend BV510 Mouse Anti-Human CD8, Clone: RPA-T8), CD45RA (Biolegend: 304 136, Biolegend BV650 Mouse Anti-Human CD45RA, Clone: HI100). Blocking of Fc receptors was achieved using the Human TruStain FcX (BioLegend). For the detection of specific intracellular markers (Foxp3 (Thermo Fisher Scientific: 17-4777-42, Thermo Fisher Scientific APC Mouse Anti-Human Foxp3, Clone: 236A/E7)), cells were treated with the Foxp3/Transcription Factor Staining Buffer Kit (Thermo Fisher Scientific).

Flow cytometry analysis was conducted on instruments including the LSR Fortessa (BD Biosciences), NovoCyte 3000 Flow Cytometer, and NovoCyte Quanteon Flow Cytometer (both from Agilent, Santa Clara, CA). Data acquisition and subsequent interpretations were performed utilizing the FlowJo software (BD Biosciences).

The cell fractions were defined as follows: naive Tconv: CD45^+^ CD3^+^ CD4^+^ Foxp3^−^ CD45RA^+^; naive CTL: CD45^+^ CD3^+^ CD8^+^ CD45RA^+^; naive Treg: CD45^+^ CD3^+^ CD4^+^ Foxp3^+^ CD45RA^+^; memory Tconv: CD45^+^ CD3^+^ CD4^+^ Foxp3^−^ CD45RA^−^; memory CTL: CD45^+^ CD3^+^ CD8^+^ CD45RA^−^; activated Treg: CD45^+^ CD3^+^ CD4^+^ Foxp3^+^ CD45RA^+^.

### Cell quantifications based on histopathological images

The specimens were scanned using a whole-slide imaging scanner (Hamamatsu NanoZoomer 2.0HT; Hamamatsu Photonics K.K., Hamamatsu, Japan) with a 20× objective lens. Hematoxylin and eosin (HE) stained images captured at a 40× magnification were subsequently resized to achieve a resolution of 0.25 μm per pixel. Each image was then segmented into 225 equal sections, and cell segmentation was carried out using a self-organizing map (SOM) algorithm. For this purpose, the Seg-SOM algorithm was employed in this study. Lymphocyte counts were then quantified in the segmented images.

### CNA analysis

We calculated the Tumor fraction based on Copy Number Alterations (CNA) using ichorCNA (31), which utilizes a hidden Markov model (HMM). Initially, we computed read counts at 1Mb intervals using readCounter with parameters (–window 1 000 000 –quality 20). Subsequently, CNA identification and Tumor fraction detection were performed using runIchorCNA.R. The parameters used were –ploidy ‘c(2,3)’ –normal ‘c(0.5,0.6,0.7,0.8,0.9)’ –maxCN 5 –includeHOMD False –chrs ‘c(1:22, ’X‘)’ –chrTrain ‘c(1:22)’ –estimateNormal True –estimatePloidy True –estimateScPrevalence True –scStates ‘c(1,3)’ –txnE 0.9999 –txnStrength 10 000. The reference was downloaded from https://github.com/broadinstitute/ichorCNA/tree/master/inst/extdata.

### Analysis of ICC samples

We investigated 72 patients with ICC who underwent surgery at the Kyoto University Hospital between 2004 and 2016. The characteristics of the participants are summarized in Supplementary Table S3. We performed cell deconvolution using MEnet. To profile the ICC samples, we decomposed the cell frequency matrix, which was adapted with min-max scaling using NMF. For the computation of NMF, we utilized the Python package scikit-learn (0.24.1). For survival analysis and the calculation and visualization of the concordance index, we utilized the Python package lifelines (0.27.0). For the visualization of Spearman correlation, the R package corrplot was used.

## Results

### The MEnet algorithm

**Figure 1.**
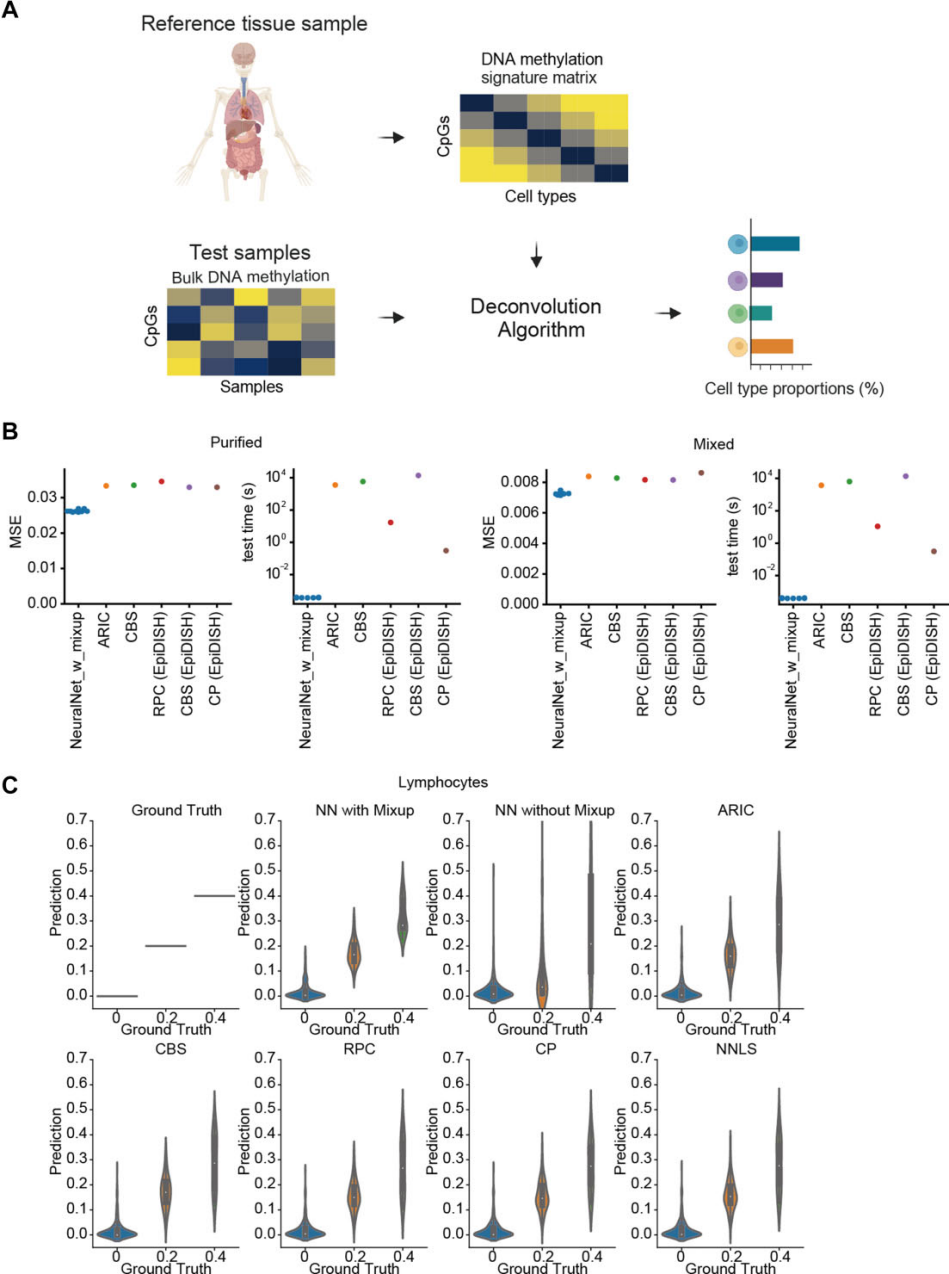
Model optimization for DNA methylation-based cell deconvolution. (**A**) Schematic representation of the deconvolution method utilizing DNA methylation. Cell proportions of the test samples were predicted by a deconvolution algorithm with the DNA methylation signature matrix of cells (Created using Biorender.com). (**B**) The panels represent the mean squared error (MSE) and calculation time of the prediction by each method for purified cells or an artificially mixed dataset. Refer to Supplementary Figure S1 for additional details. (**C**) Violin plots represent lymphocyte counts, comparing ground truth with predictions made by neural network (NN) + mixup and other existing tools. (Refer to Supplementary Figure S2 for additional details.)

Firstly, we attempted to select an optimal method for cell deconvolution using DNA methylation. We trained and tested models using DNA methylation datasets from the databases (ENCODE, Roadmap, Blueprint and the human methylome atlas) (Figure 1A). Differentially methylated regions were selected for each sample and used for the training. In addition to the non-negative least squares, which are widely used in deconvolution using DNA methylation, we prepared models based on common machine learning techniques such as random forest, support vector regression, gradient boosting and neural network (NN) models. NN offers the advantage of making complex non-linear predictions and the flexibility of adding data augmentation. While conventional reference-based deconvolution uses only purified cells as references, real-world datasets often consist of a mixture of multiple cell types, which may not be suitable for the training. To adapt this, the augmentation technique of randomly mixing the training dataset, mixup, is commonly used in deep learning (32) and is known to outperform existing methods in RNAseq deconvolution (33). Therefore, we also prepared a model that added mixup to the NN. The NN model recorded the highest accuracy for both purified cells and mixed data (Mean Squared Error 0.0265 in NN + mixup, 0.0263 in NN, 0.0320–0.0557 in other methods for purified cells, and 0.00730 in NN + mixup, 0.0161 in NN, 0.0842–0.0182 in other methods for mixed cells in average), and the NN model was also superior in the computation time for the test dataset (Supplementary Figure S1A, B). While the prediction accuracy for purified cells was highest for the NN without mixup, the NN with mixup showed significantly improved accuracy for mixed cells compared to those without mixup. This suggests that adding mixup to NN can provide more accurate estimation via suppressing overfitting, a pronounced problem of the NN model. On the other hand, the training computation time was the longest for the NN + mixup model. When comparing the architecture of NN + mixup with existing deconvolution methods, NN + mixup showed better accuracy than ARIC (17), CP/QP (27), CIBERSORT (18), RPC (16) for both purified and mixed cells (Figure 1B). For instance, in identifying cell populations mixed with B cells, CD4^+^ T cells, CD8^+^ T cells, and NK cells at similar proportions, MEnet was able to compute the proportions with less error than other methods (Figure 1C, Supplementary Figure S2). In terms of computation time, while NN + mixup required longer training time, the prediction time was over 781 times faster than that of other existing methods (Supplementary Figure S1C). This demonstrated that the NN + mixup possesses high performance in estimating cell population frequencies from complex samples.

### Development of MEnet

**Figure 2.**
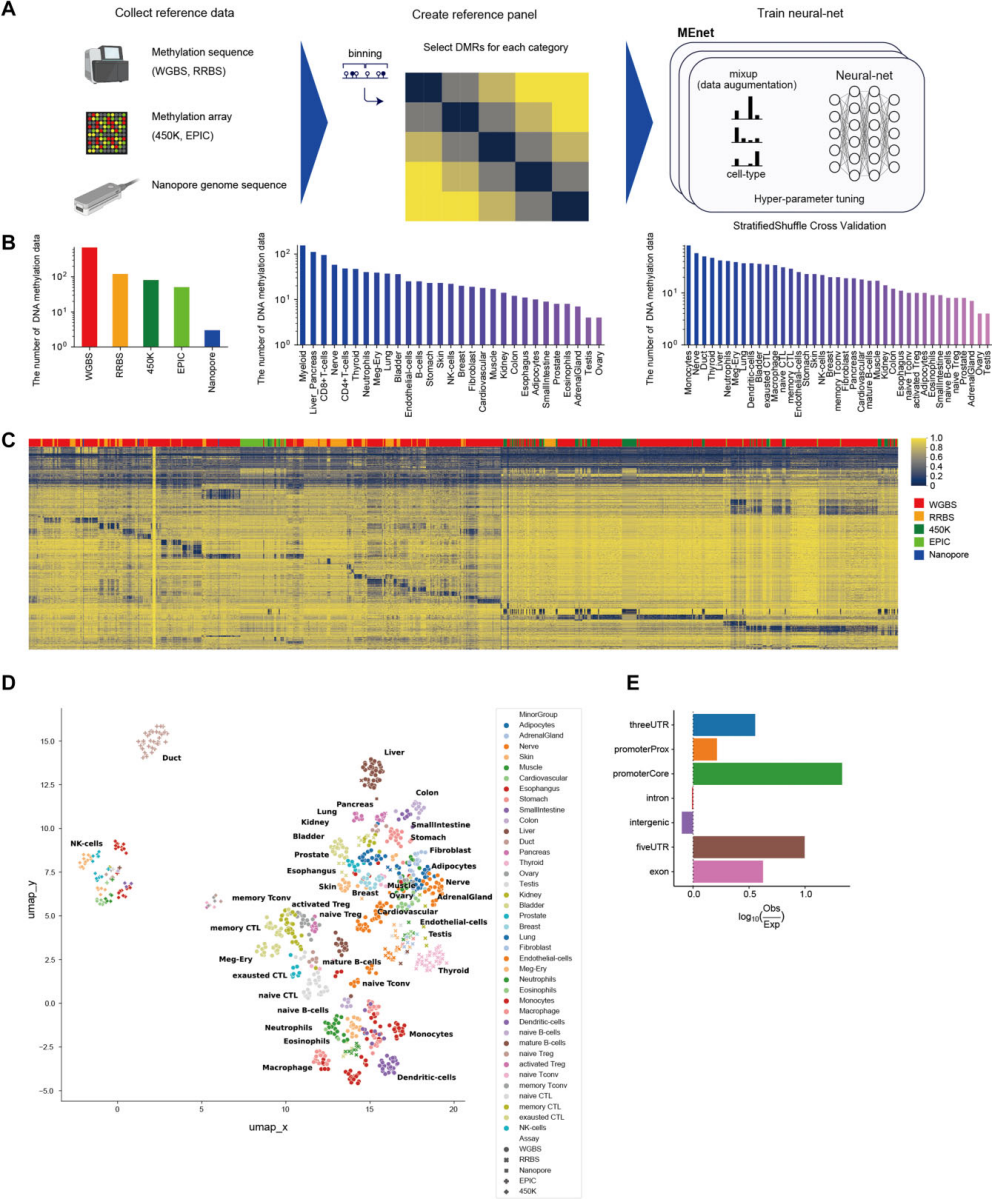
MEnet architecture and cell feature regions for its prediction. (**A**) Schematic representation of the training dataset for MEnet (Created using Biorender.com). (**B**) Bar plots show the composition of reference samples: methods for DNA methylation analysis (left), major category (middle), and minor category (right). (**C**) Heatmap showing mean DNA methylation rate of the feature regions for all reference samples. X-, Y-axes and color indicate samples, regions, and the DNA methylation rate, respectively. The top row indicates the methods for DNA methylation analysis. (**D**) UMAP visualization of the reference samples based on DNA methylation rates in the feature regions. The plot displays the key cell populations identified in the dataset, with cell labels based on their cell surface markers or origins. (**E**) Bar plot shows the enrichment of the feature regions in the genome.

Based on the optimal method for cell deconvolution determined above, we sought to develop a new deconvolution framework for analyzing tumor microenvironment in cancer patients. NN models have many hyperparameters, such as the number of layers and the number of nodes. Applying cross-validation and Bayesian optimization to determine the parameters, we developed a cell deconvolution tool based on the DNA methylation by employing NN + mixup, and named it MEnet (Figure 2A).

### Expansion of training data

To achieve effective deconvolution, we expanded the amount and the diversity of the training data by adding original data (samples derived from PBMCs and surgically removed tumors) and curated data (4,12,34–64) to the dataset consisting of ENCODE, Roadmap, Blueprint, and the human methylome atlas (Supplementary Tables S1 and S2). Importantly, from the surgically removed tumors, we isolated tumor-infiltrating immune cells with different states, such as exhausted CD8^+^ T, activated CD8^+^ T, naive Treg and effector Treg cells and added their whole genome bisulfite sequencing (WGBS) data to the dataset to improve performance in profiling the tumor immune microenvironment. The total number of genome-wide DNA methylation data collected reached 945 (Figure 2B). These samples are categorized into two layers: Major group and Minor group, with 29 and 39 categories, respectively.

To generalize the model to various assays, our reference includes not only WGBS data but also those from reduced representation bisulfite sequencing (RRBS), DNA methylation microarrays, and nanopore sequencers (Figure 2B). We calculated 1 000 bp bins across the entire genome and selected up to 2 000 characteristic regions for each cell type, totaling 87 726 regions (Figure 2C, D, Supplementary Figure S3A). The feature regions were enriched in the promoter core region (log_10_Observed/Expected = 1.33, *P*= 1.72 × 10^−206^), followed by 5′UTR (log_10_Observed/Expected = 1.00, *P*< 10^−100^) and exon (log_10_Observed/Expected = 0.62, *P*< 10^−100^) (Figure 2E, Supplementary Figure S3B).

### Training and optimization of MEnet

Using this reference dataset, we trained MEnet for the Minor group through 6-fold cross-validation with Bayesian Optimization (Supplementary Figure S4A-C). After >3000 trials, including the epochs where early stopping occurred, we determined an optimal architecture with two hidden layers and a hidden dimension of 1030, along with the model weights. MEnet attained an improved computational speed by GPU in addition to parallel computation through distributed learning, and could efficiently train on large datasets using cloud computing.

### Evaluation of sequencing depth requirements

**Figure 3.**
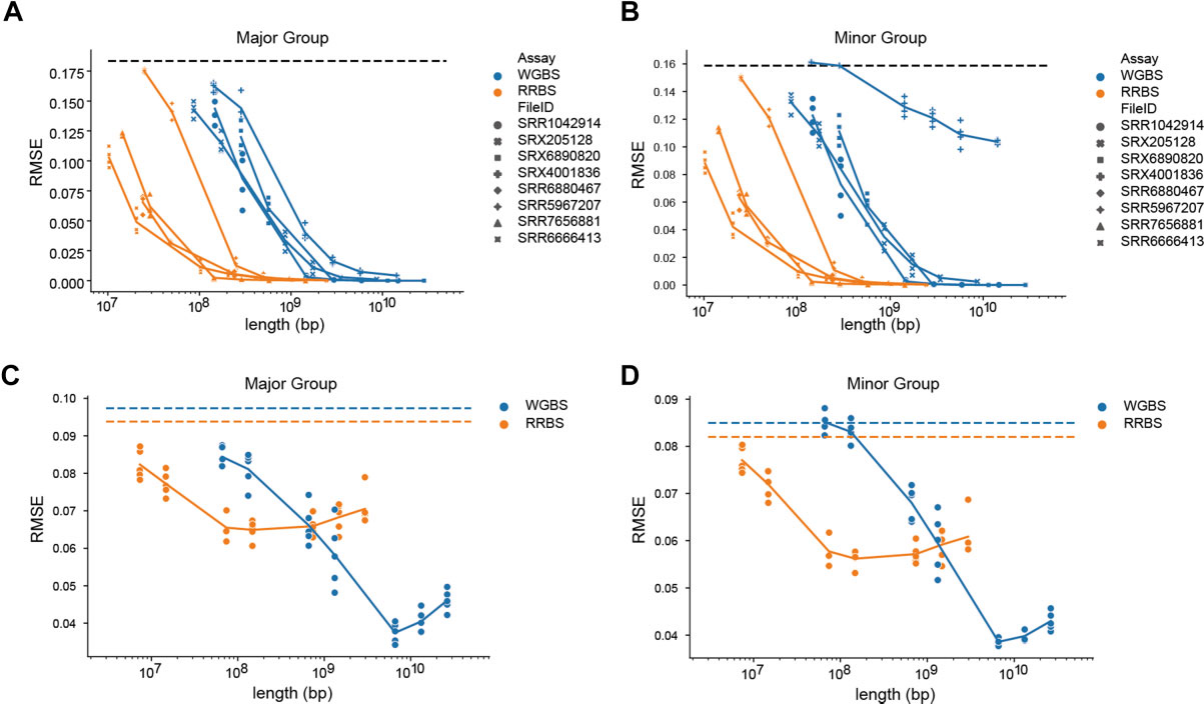
Subsampling analysis for determining the required read depth for the prediction. Root mean squared error (RMSE) of the prediction for subsampled reads from whole genome bisulfite sequencing (WGBS) and reduced representation bisulfite sequencing (RRBS) samples not used in model training. (**A**) and (**B**) represent the results of subsamples using purified cell data, while (**C**) and (**D**) depict the results of simulated mixture data. (A) and (C) show the results at the Major Group level, and (B) and (D) at the Minor Group level. Various proportions of sequence reads are sampled with five independent seeds and evaluated for accuracy. The dotted line across the panels represents the average RMSE for 500 random predictions. Markers represent independent samples (A and B).

Next, we evaluated the sequencing depth required for MEnet to predict cell proportions. We assessed accuracy using eight distinct datasets of purified cell data from WGBS or RRBS that were not used for training, as well as mixed data from these datasets (Figure 3A–D). For the purified data, the root means square error (RMSE) of RRBS saturated at approximately 2 × 10^8^ bp, while the RMSE of WGBS did at 2 × 10^9^ bp. For the mixed data, the RMSE of RRBS saturated at about 1 × 10^8^ bp, and the RMSE of WGBS at 7 × 10^9^ bp. We found that RRBS required about ten times less data than WGBS. This is likely because RRBS targets a limited number of methylation sites. In addition, satisfactory accuracy could be achieved with less than 3x genome coverage, even for WGBS data. In this simulation, predictions using RRBS data had inferior accuracy compared to those using WGBS data, especially for the mixed dataset, at both minor and major group resolutions. In addition, MEnet achieved lower RMSE values compared to these other methods, including, ARIC, CIBERSORT, RPC and CP/QP (Supplementary Figure S5A-D).

### Benchmarking MEnet

**Figure 4.**
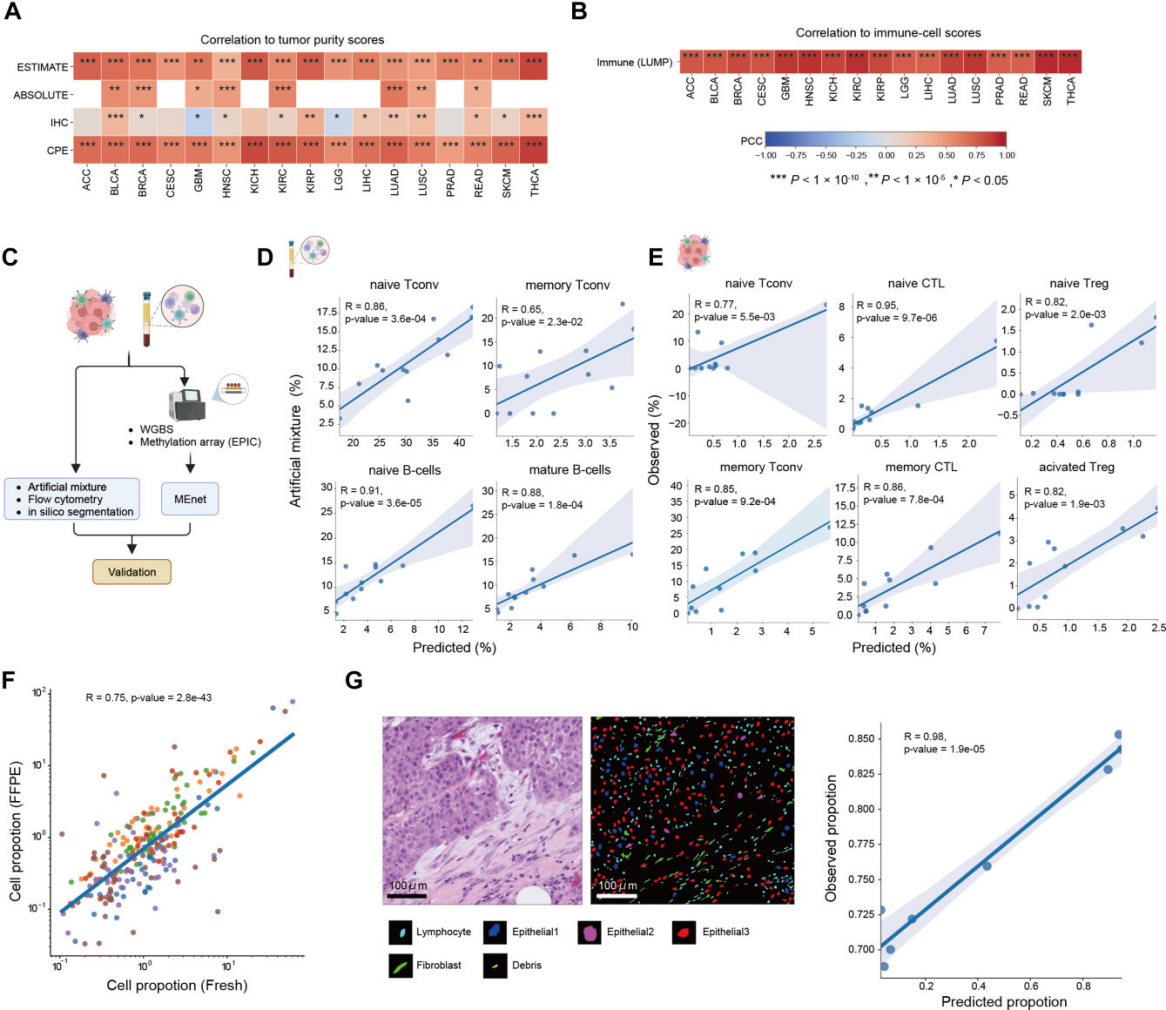
Evaluation of the performance of MEnet. (**A**) Systematic validation of the MEnet-derived tumor purity scores in the corresponding Illumina 450k DNA methylation datasets from TCGA (ACC: adrenocortical carcinoma, BLCA: bladder urothelial carcinoma, BRCA: breast invasive carcinoma, CESC: cervical squamous cell carcinoma and endocervical adenocarcinoma, GBM: glioblastoma multiforme, HNSC: head and neck squamous cell carcinoma, KICH: kidney chromophobe, KIRC: kidney renal clear cell carcinoma, KIRP: kidney renal papillary cell carcinoma, LGG: brain lower grade glioma, LIHC: liver hepatocellular carcinoma, LUAD: lung adenocarcinoma, LUSC: lung squamous cell carcinoma, PRAD: prostate adenocarcinoma, READ: rectum adenocarcinoma, SKCM: skin cutaneous melanoma, THCA: thyroid carcinoma). The heatmap depicts the Pearson correlation coefficients (PCC) between the tumor purity estimated using MEnet and the tumor purity estimated with different methods, including ESTIMATE, ABSOLUTE (CNV-based), immunohistochemistry (IHC), and consensus purity estimate (CPE). Asterisks indicate statistical significance level (*P* value thresholds) as shown in the figure. *P* values were derived from a one-tailed correlation test. (**B**) The heatmap depicts the PCC between the total immune cell fraction estimated using MEnet and the corresponding total immune cell fraction obtained by other methods (LUMP: the DNA methylation-based leukocyte unmethylation for purity). Asterisks indicate statistical significance level (*P* value thresholds) as shown in panel B. *P* values were derived from a one-tailed correlation test. (**C**) Schematic view for evaluating the performance of MEnet (Created using Biorender.com). (**D**) Scatter plot comparing the proportion of mixed immune cells (y-axis) and the proportion of immune cells predicted by MEnet (x-axis). The prediction was performed against artificially mixed cell DNA of sorted purified peripheral blood mononuclear cells (PBMCs) (GSE167998) (Created using Biorender.com). (**E**) Scatter plots comparing immune cell proportion in tumor tissues measured by flow cytometry (y-axis) and those predicted by MEnet (x-axis). *n* = 11 (Created using Biorender.com). (**F**) Scatter plot comparing the proportions of various cell subsets estimated from formalin-fixed paraffin-embedded (FFPE) sample-derived DNA (y-axis) versus those estimated from fresh sample-derived DNA (x-axis). DNA was extracted and analyzed from both FFPE and fresh preparations of the same skin cancer samples (six donors). Each point represents a cell subset from an individual sample. Colors indicate the individuals (Red, Blue, Orange, Purple and Green are melanoma. Brown is squamous cell carcinoma). (**G**) Comparison between results by MEnet and cell-type inference from Hematoxylin and eosin (HE) sections. HE staining of melanoma cancer tissue section (left), and the cell classes inferred by Seg-SOM pipeline (center). As demonstrated in the Seg-SOM study, the three epithelial cell types are distinguished based on their nuclear morphology. The right panel shows a scatter plot comparing the proportion of lymphocyte class nuclei estimated by the Seg-SOM pipeline (y-axis) and the proportion of lymphocytes estimated by MEnet (x-axis). *n* = 8.

To validate the accuracy of MEnet, we first applied our method to The Cancer Genome Atlas (TCGA) data, where pathological estimates of tumor and immune fractions are available. We compared the tumor and immune cell scores calculated by MEnet with those obtained from different estimation methods, such as ESTIMATE, ABSOLUTE and LUMP, as previously described (65). The results showed significant positive correlations between MEnet's predictions and the reference values for both tumor and immune cell scores across multiple cancer types (Figure 4A, B). These findings demonstrate the robustness and reliability of MEnet in estimating tumor purity and immune cell infiltration in real-world cancer datasets.

We further evaluated the performance of MEnet using various real-world datasets (Figure 4C). A primary concern was the predictive accuracy of MEnet, especially to those related to cellular characteristics such as activation states. Initially, we used 12 types of artificial mixtures consisting of purified cells (over 90% purity by cell sorter) from human PBMCs (12), to compare MEnet-predicted values with actual values. We found a significant positive correlation between the two values regarding not only broad cell subsets but also finer classifications, including cellular states (Figure 4D, Supplementary Figure S6A). Next, using tumor samples (ovary, stomach, skin and colon cancers), we further analyzed the specific states of immune cells, especially the frequencies of activated, naive, or exhausted T cells. By comparing the cell frequency by flow cytometry with those by MEnet calculated from WGBS data, we observed some differences in correlation patterns across different cell subsets. In the lymphoid lineage, a strong correlation was evident, even when considering detailed cell states such as activated CD8^+^ T cells (Figure 4E). However, the myeloid lineage showed a weak correlation, potentially due to the decrease in cell numbers caused by cell damage during the cell dissociation process (Supplementary Figure S6B). Despite these variations, the overall alignment was consistent when looking at specific classifications, indicating the validity of MEnet-mediated cell subset prediction even in cancer tissues.

To determine the feasibility of using sections after pathological diagnosis, we examined whether DNA methylation data from formalin-fixed paraffin-embedded (FFPE) sections is suitable for MEnet. Using skin cancer samples and performing WGBS on both FFPE and fresh tissues, we observed a strong correlation in cell frequencies predicted by MEnet between them (Figure 4F). Additionally, to determine the consistency between MEnet and the standard histopathological method for FFPE sections, we compared the lymphocyte frequencies estimated by MEnet with those determined from HE-stained section images. For the HE-stained sections, we employed Seg-SOM (66), a computational vision map based on an artificial neural network, to identify and classify cell types. Seg-SOM was trained on a comprehensive dataset of expertly annotated cell types and has been validated for accurate cell type identification in various tissues. The comparison between MEnet and Seg-SOM-based cell type identification in HE-stained sections confirmed the consistency between the two methods (Figure 4G).

Lastly, we explored the possibility of predicting cell frequency using DNA methylation data from direct DNA sequencing. We sequenced DNA from activated Treg, activated conventional CD4^+^ T (Tconv), and naive Tconv cells by nanopore sequencers and retrieved DNA methylation data from the sequencing signals. The predicted cell frequencies by MEnet were consistently matched to the original cell types (Supplementary Figure S6C). Overall, these results demonstrated the reliability of MEnet and its potential in various clinical applications.

### Exploring the heterogeneity of cfDNA

**Figure 5.**
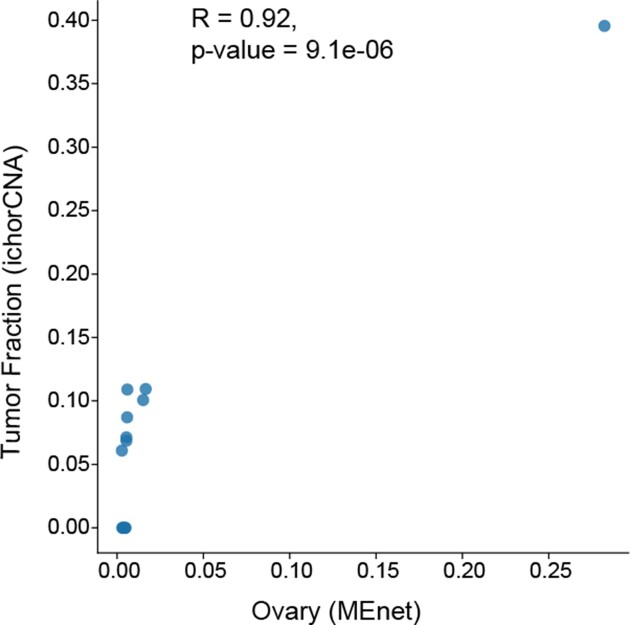
Cell-free DNA analysis for detecting tumors by MEnet. Scatter plot showing a correlation between ovary ratio predicted by MEnet (x-axis) and tumor ratio inferred by copy number alternation (CNA) (y-axis) in cell-free DNA from ovarian cancer patients (*n* = 13). Cell-free DNAs were isolated from the peripheral blood of ovarian cancer patients and subjected to DNA methylation analysis.

We next evaluated the potential clinical application of MEnet for cfDNA derived from peripheral blood of cancer patients. We performed WGBS on cfDNA from 13 ovarian cancer patients, applied MEnet, and obtained their deconvoluted cell profiles. The cell population corresponding to the origin of cancer (ovary) by MEnet was compared with the tumor fraction inferred from genomic copy number alternation (CNA) (31). A significant correlation was observed between the tumor fraction percentages determined by CNA and those by MEnet (Figure 5). These results suggest the potential of MEnet for a wide range of clinical applications, e.g. for assessing the progression of cancer, selecting individualized treatment methods, and monitoring therapeutic efficacy.

### The heterogeneity in intrahepatic cholangiocarcinoma

**Figure 6.**
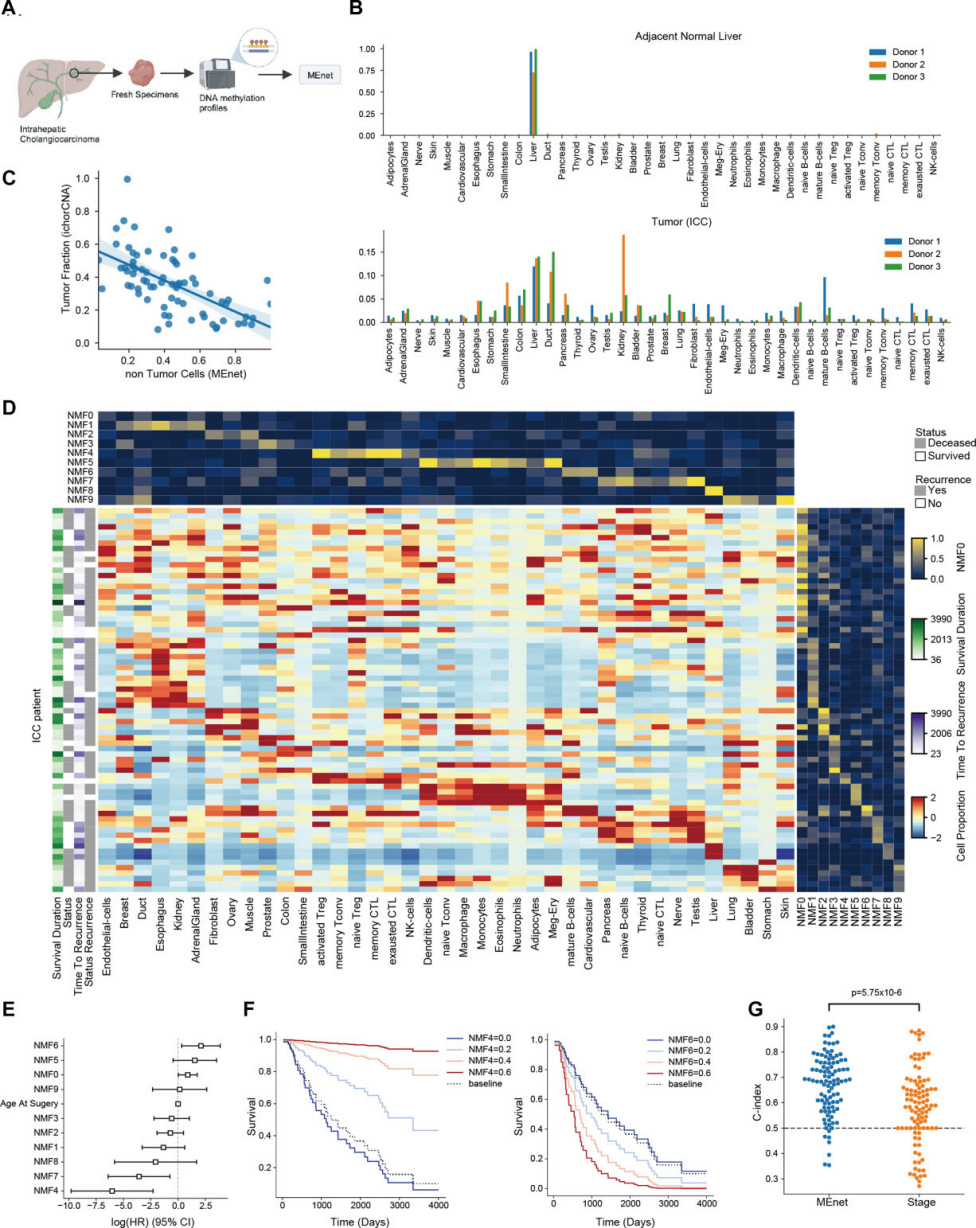
Cell profiling of 72 ICC samples predicted by MEnet. (**A**) An overview of the intrahepatic cholangiocarcinoma (ICC) sample analysis. (**B**) Bar plots showing the deconvolution results for the non-tumor sites (upper) and tumor sites (lower) in same ICC patients (*n* = 3) (Created using Biorender.com). (**C**) Scatter plot showing the tumor proportion predicted by CNA (y-axis) and the proportion of non-tumor cell fractions estimated by MEnet (x-axis). Non-tumor cells were calculated by the total values of blood cells, liver, fibroblast, adipocyte, endothelial cells, cardiovascular, and muscle. *R* = –0.61, *P*= 1.5 × 10^−6^. (**D**) Heatmap showing the cell frequency profile and clinical prognosis of 72 patients. It displays the cell frequency normalized by cell type (center), cell features decomposed by NMF (upper), sample features (right), and prognosis (left). Prognosis involves survival (status), recurrence (status recurrence), survival duration, and time to recurrence. (**E**) Forest plot of the Cox-Hazard analysis for survival duration. The analysis incorporates NMFs and cancer stages (quantified as integers from 1 to 4) as covariates. (**F**) NMF4 or NMF6 is associated with good or poor survival duration, respectively. Kaplan-Meier plots for varying covariates of NMF4 (left) and NMF6 (right) are indicated. (**G**) Survival duration can be estimated by the cell proportion predicted by MEnet. Shown are distributions of the Concordance-index (C-index) for survival duration prediction by MEnet (MEnet) or cancer stage classification (Stage), calculated by bootstrap. Bootstrap involved performing Cox hazard regression on 62 randomly selected samples within 72 samples and calculating the *C*-index on the remaining 10 samples, which are repeated 100 times. The distribution of the *C*-index calculated from MEnet results and cancer stage classification was tested using the Mann–Whitney *U*-test.

We next evaluated the scalability and reliability of MEnet in clinical applications using surgically removed samples of intrahepatic cholangiocarcinoma (ICC) (Figure 6A). First, we compared an ICC tumor tissue with its adjacent normal liver sample in the same patient. By analyzing their DNA methylation data obtained by nanopore direct DNA sequencing, normal liver sites were primarily predicted to consist of liver tissue (Figure 6B). In contrast, in ICC tumor sites, the proportion of liver was decreased, while the proportions of duct, pancreas, and intestine were increased (Figure 6B). Since ICC is a cancer originating from the bile duct and not hepatocytes (67), the increase of these non-liver endoderm tissues is reasonable, and the rise in other digestive tissues might reflect transformations those cancers are undergoing. Interestingly, abundant immune cells were only observed in cancer sites, suggesting the feasibility of MEnet in profiling the tumor microenvironment.

Subsequently, to investigate the correlation between the diversity of ICC and its tumor microenvironment, we collected tumor samples from 72 ICC patients and their medical records spanning a decade. We determined the DNA methylation states of the tumors using WGBS and explored the association between the deconvoluted cellular profiles and clinical outcomes, such as survival and recurrence. Initially, the tumor proportion estimated by CNA and the total proportion of non-tumor cells by MEnet (i.e. the sum of fibroblast, liver, muscle, etc.) showed a significant negative correlation (Figure 6C, *r* = −0.61, *P*= 1.5 × 10^−6^). We then examined the correlation between clinical information and the predicted cell frequencies (Supplementary Figure S7A). The cell frequencies by deconvolution showed only limited correlations with cancer stage, tumor diameter, blood markers for liver function, and tumor markers, such as carcinoembryonic antigen (CEA) and CA19-9. It suggests that the cellular profiles within the tumor are independent of these indicators.

Next, to investigate the correlation between predicted cell profiles and clinical progressions, we decomposed the profiles of the 72 ICC cases and 39 cell types into several patterns (Figure 6D). Using a non-negative feature matrix (NMF), we decomposed the cell frequency profiles for each patient into ten components (Figure 6D). This approach not only simplifies the analysis by reducing the number of variables but also effectively overcomes the challenges of multicollinearity, a common issue when multiple cell types exhibit correlated behaviors. Examining the association between these ten components and prognosis, we found that higher NMF4 or NMF7 indicated better outcomes, while higher NMF6 indicated worse outcomes (Figure 6E). NMF4 had high weights for activated Tconv, exhausted cytotoxic T lymphocyte (CTL), and memory Treg cells, suggesting that abundant immune cells are infiltrating into tumors with high immunogenicity (Figure 6F). These immunological features may be related to better overall survival. NMF6 was dominated by cardiovascular, hinting at an inverse correlation between vascular invasion and prognosis (Figure 6F). While the association of NMF with recurrence was less pronounced than with overall survival, it was evident that higher NMF4 led to a lower likelihood of recurrence. Furthermore, when we examined the potential for prognosis prediction based on intratumoral cell profiles using bootstrap simulation, we could predict the overall survival based on the NMF calculated from the cell population. The prediction accuracy surpassed predictions based on the currently used cancer stage classification (Figure 6G, *P*= 5.75 × 10^−6^). The NMF-based prediction was also able to predict the time to recurrence, yet the stage classification was better (*P*= 0.00401, Supplementary Figure S8A, B). Altogether, these results demonstrate the feasibility of predicting cellular profiles by MEnet and its application in clinical settings.

## Discussion

In this study, we developed a deconvolution tool based on DNA methylation information, termed MEnet, and applied it to cfDNA from blood and ICC tumor tissues, verifying its clinical significance. Compared to the previous methods, neural networks enhanced robustness through abundant parameters and data augmentation in combination with large datasets, achieving high accuracy of the tool. The utility of MEnet was evaluated not only using commonly utilized peripheral blood samples but also with samples derived from tissues, cfDNAs, FFPE sections and tumors, demonstrating its robustness across diverse scenarios. MEnet showed prominent potential in clinical applications, potentially leading to personalized treatments. In addition, to the best of our knowledge, our model is the only one constructed across various platforms, including nanopore DNA sequencing, WGBS, RRBS and methylation arrays, suggesting its potential for flexible clinical applications.

The complexity of the parameter settings of neural networks for cell deconvolution would be a major obstacle to using them. MEnet addressed this issue by Bayesian optimization for architecture optimization. In addition, with respect to the enormous calculation time and computational resources needed for the training, we have resolved these issues by GPU computing and distributed computing. Meanwhile, with the emergence of data from single-cell WGBS (68–71) or methylation data inferred from single-cell RNAseq (65), this model is also designed to be flexible enough to accommodate new data. This flexibility allows for the creation of customized references for specific tissues and cancer types, enhancing its applicability across various research and clinical settings. Considering the difficulty for individual users to prepare and calculate a large volume of datasets, we expect most users to operate with the pre-trained datasets we established.

One potential application of MEnet is the analysis of cfDNA. By applying MEnet to cfDNA, real-time assessment of treatment response and disease progression may be achieved through the detection of tumor-derived cfDNA in the blood (72). Additionally, with the observed changes in peripheral immune cells associated with immune-related adverse events during immune checkpoint inhibitor treatment (73,74), the ability of MEnet to simultaneously detect immune cells in the blood may be useful for real-time detection of immune-related adverse events. The application of MEnet offers promise for more accurate treatment strategies and enhances the efficacy and safety of cancer immunotherapies.

In this study, we also demonstrated the efficacy of cell profiling in a large dataset of ICC. Historically, ICC has been categorized into two subclasses: inflammation and proliferation subclasses, with the inflammation subclass being shown to have a better prognosis (75). The high correlation of a favorable outcome of ICC with the extracted feature NMF4, representing expanded tumor-infiltrating lymphocytes, was consistent with this classification. Beyond this, the identification of several tumor microenvironment programs brought us closer to a comprehensive understanding of ICC. Given the absence of tumor markers specific to ICC, specific cell monitoring using DNA methylation might also hold promise.

However, it is important to note that the absolute values calculated by MEnet may deviate for certain subsets, such as naïve Tconv and memory Tconv. Therefore, caution should be exercised when considering the direct application of MEnet for clinical purposes. Despite these discrepancies, the relative abundances within each cell type are well-correlated, suggesting that MEnet remains suitable for comparative analyses within a given cell type.

In summary, cellular deconvolution through DNA methylation using MEnet has the potential to emerge as a new analytical strategy for clinical samples.

## Supplementary Material

zcae022_Supplemental_Files

## Data Availability

Raw sequence data have been deposited at JGA via the NBDC human database (Accession Number: JGAS000676) under controlled access. TCGA Illumina 450k datasets for ACC, BLCA, BRCA, CESC, GBM, HNSC, KICH, KIRC, KIRP, LGG, LIHC, LUAD, LUSC, PRAD, READ, SKCM and THCA datasets are available from the GDC data portal https://portal.gdc.cancer.gov/. The source code for MEnet and the trained model are provided on Github (https://github.com/yyoshiaki/MEnet) and Zenodo (https://doi.org/10.5281/zenodo.11085605). Trained weights for MEnet are available on Figshare (https://figshare.com/s/9a3b8d9b6b507aa32f78). The scripts used to generate the figures in this manuscript are available on GitHub (https://github.com/yyoshiaki/MEnet_manuscript) and Zenodo (https://doi.org/10.5281/zenodo.11085611).

## References

[1] Greenberg M.V.C., Bourc’his D. The diverse roles of DNA methylation in mammalian development and disease. Nat. Rev. Mol. Cell Biol. 2019; 20:590–607. 31399642 10.1038/s41580-019-0159-6

[2] Shalek A.K., Satija R., Adiconis X., Gertner R.S., Gaublomme J.T., Raychowdhury R., Schwartz S., Yosef N., Malboeuf C., Lu D. et al. Single-cell transcriptomics reveals bimodality in expression and splicing in immune cells. Nature. 2013; 498:236–240. 23685454 10.1038/nature12172 PMC3683364

[3] Loyfer N., Magenheim J., Peretz A., Cann G., Bredno J., Klochendler A., Fox-Fisher I., Shabi-Porat S., Hecht M., Pelet T. et al. A DNA methylation atlas of normal human cell types. Nature. 2023; 613:355–364. 36599988 10.1038/s41586-022-05580-6 PMC9811898

[4] Ohkura N., Yasumizu Y., Kitagawa Y., Tanaka A., Nakamura Y., Motooka D., Nakamura S., Okada Y., Sakaguchi S. Regulatory T cell-specific epigenomic region variants are a key determinant of susceptibility to common autoimmune diseases. Immunity. 2020; 52:1119–1132. 32362325 10.1016/j.immuni.2020.04.006

[5] Moran S., Martínez-Cardús A., Sayols S., Musulén E., Balañá C., Estival-Gonzalez A., Moutinho C., Heyn H., Diaz-Lagares A., de Moura M.C. et al. Epigenetic profiling to classify cancer of unknown primary: a multicentre, retrospective analysis. Lancet Oncol. 2016; 17:1386–1395. 27575023 10.1016/S1470-2045(16)30297-2

[6] Chakravarthy A., Furness A., Joshi K., Ghorani E., Ford K., Ward M.J., King E.V., Lechner M., Marafioti T., Quezada S.A. et al. Pan-cancer deconvolution of tumour composition using DNA methylation. Nat. Commun. 2018; 9:3220. 30104673 10.1038/s41467-018-05570-1 PMC6089972

[7] Andargie T.E., Tsuji N., Seifuddin F., Jang M.K., Yuen P.S., Kong H., Tunc I., Singh K., Charya A., Wilkins K. et al. Cell-free DNA maps COVID-19 tissue injury and risk of death and can cause tissue injury. JCI Insight. 2021; 6:1443. 10.1172/jci.insight.147610 PMC8119224 33651717

[8] Zemmour H., Planer D., Magenheim J., Moss J., Neiman D., Gilon D., Korach A., Glaser B., Shemer R., Landesberg G. et al. Non-invasive detection of human cardiomyocyte death using methylation patterns of circulating DNA. Nat. Commun. 2018; 9:1443. 29691397 10.1038/s41467-018-03961-y PMC5915384

[9] Murata H., Kinoshita M., Yasumizu Y., Motooka D., Beppu S., Shiraishi N., Sugiyama Y., Kihara K., Tada S., Koda T. et al. Cell-free DNA derived from neutrophils triggers type 1 interferon signature in Neuromyelitis Optica spectrum disorder. Neurol. Neuroimmunol. Neuroinflamm. 2022; 9:3. 10.1212/NXI.0000000000001149 PMC8874356 35210295

[10] Havel J.J., Chowell D., Chan T.A. The evolving landscape of biomarkers for checkpoint inhibitor immunotherapy. Nat. Rev. Cancer. 2019; 19:133–150. 30755690 10.1038/s41568-019-0116-x PMC6705396

[11] Katsman E., Orlanski S., Martignano F., Fox-Fisher I., Shemer R., Dor Y., Zick A., Eden A., Petrini I., Conticello S.G. et al. Detecting cell-of-origin and cancer-specific methylation features of cell-free DNA from Nanopore sequencing. Genome Biol. 2022; 23:158. 35841107 10.1186/s13059-022-02710-1 PMC9283844

[12] Salas L.A., Zhang Z., Koestler D.C., Butler R.A., Hansen H.M., Molinaro A.M., Wiencke J.K., Kelsey K.T., Christensen B.C. Enhanced cell deconvolution of peripheral blood using DNA methylation for high-resolution immune profiling. Nat. Commun. 2022; 13:761. 35140201 10.1038/s41467-021-27864-7 PMC8828780

[13] Moss J., Magenheim J., Neiman D., Zemmour H., Loyfer N., Korach A., Samet Y., Maoz M., Druid H., Arner P. et al. Comprehensive human cell-type methylation atlas reveals origins of circulating cell-free DNA in health and disease. Nat. Commun. 2018; 9:5068. 30498206 10.1038/s41467-018-07466-6 PMC6265251

[14] Houseman E.A., Kile M.L., Christiani D.C., Ince T.A., Kelsey K.T., Marsit C.J. Reference-free deconvolution of DNA methylation data and mediation by cell composition effects. BMC Bioinf. 2016; 17:259. 10.1186/s12859-016-1140-4 PMC4928286 27358049

[15] Koestler D.C., Jones M.J., Usset J., Christensen B.C., Butler R.A., Kobor M.S., Wiencke J.K., Kelsey K.T. Improving cell mixture deconvolution by identifying optimal DNA methylation libraries (IDOL). BMC Bioinf. 2016; 17:120. 10.1186/s12859-016-0943-7 PMC4782368 26956433

[16] Teschendorff A.E., Breeze C.E., Zheng S.C., Beck S. A comparison of reference-based algorithms for correcting cell-type heterogeneity in Epigenome-Wide Association Studies. BMC Bioinf. 2017; 18:105. 10.1186/s12859-017-1511-5 PMC5307731 28193155

[17] Zhang W., Xu H., Qiao R., Zhong B., Zhang X., Gu J., Zhang X., Wei L., Wang X. ARIC: accurate and robust inference of cell type proportions from bulk gene expression or DNA methylation data. Brief. Bioinform. 2022; 23:bbab362. 34472588 10.1093/bib/bbab362

[18] Newman A.M., Liu C.L., Green M.R., Gentles A.J., Feng W., Xu Y., Hoang C.D., Diehn M., Alizadeh A.A. Robust enumeration of cell subsets from tissue expression profiles. Nat. Methods. 2015; 12:453–457. 25822800 10.1038/nmeth.3337 PMC4739640

[19] ENCODE Project Consortium Moore J.E., Purcaro M.J., Pratt H.E., Epstein C.B., Shoresh N., Adrian J., Kawli T., Davis C.A., Dobin A. et al. Expanded encyclopaedias of DNA elements in the human and mouse genomes. Nature. 2020; 583:699–710. 32728249 10.1038/s41586-020-2493-4 PMC7410828

[20] Consortium R.E., Kundaje A., Meuleman W., Ernst J., Bilenky M., Yen A., Heravi-Moussavi A., Kheradpour P., Zhang Z., Wang J. et al. Integrative analysis of 111 reference human epigenomes. Nature. 2015; 518:317–330. 25693563 10.1038/nature14248 PMC4530010

[21] Schuyler R.P., Merkel A., Raineri E., Altucci L., Vellenga E., Martens J.H.A., Pourfarzad F., Kuijpers T.W., Burden F., Farrow S. et al. Distinct trends of DNA methylation patterning in the innate and adaptive Immune systems. Cell Rep. 2016; 17:2101–2111. 27851971 10.1016/j.celrep.2016.10.054 PMC5889099

[22] Brindley P.J., Bachini M., Ilyas S.I., Khan S.A., Loukas A., Sirica A.E., Teh B.T., Wongkham S., Gores G.J. Cholangiocarcinoma. Nat. Rev. Dis. Primers. 2021; 7:65. 34504109 10.1038/s41572-021-00300-2 PMC9246479

[23] Loeuillard E., Conboy C.B., Gores G.J., Rizvi S. Immunobiology of cholangiocarcinoma. JHEP Reports. 2019; 1:297–311. 32039381 10.1016/j.jhepr.2019.06.003 PMC7001542

[24] Krueger F., Andrews S.R. Bismark: a flexible aligner and methylation caller for Bisulfite-Seq applications. Bioinformatics. 2011; 27:1571–1572. 21493656 10.1093/bioinformatics/btr167 PMC3102221

[25] Song J., Kuan P.-F. A systematic assessment of cell type deconvolution algorithms for DNA methylation data. Brief. Bioinform. 2022; 23:bbac449. 36242584 10.1093/bib/bbac449 PMC9947552

[26] Newman A.M., Steen C.B., Liu C.L., Gentles A.J., Chaudhuri A.A., Scherer F., Khodadoust M.S., Esfahani M.S., Luca B.A., Steiner D. et al. Determining cell type abundance and expression from bulk tissues with digital cytometry. Nat. Biotechnol. 2019; 37:773–782. 31061481 10.1038/s41587-019-0114-2 PMC6610714

[27] Houseman E.A., Accomando W.P., Koestler D.C., Christensen B.C., Marsit C.J., Nelson H.H., Wiencke J.K., Kelsey K.T. DNA methylation arrays as surrogate measures of cell mixture distribution. BMC Bioinf. 2012; 13:86. 10.1186/1471-2105-13-86 PMC3532182 22568884

[28] Yoshihara K., Shahmoradgoli M., Martínez E., Vegesna R., Kim H., Torres-Garcia W., Treviño V., Shen H., Laird P.W., Levine D.A. et al. Inferring tumour purity and stromal and immune cell admixture from expression data. Nat. Commun. 2013; 4:2612. 24113773 10.1038/ncomms3612 PMC3826632

[29] Carter S.L., Cibulskis K., Helman E., McKenna A., Shen H., Zack T., Laird P.W., Onofrio R.C., Winckler W., Weir B.A. et al. Absolute quantification of somatic DNA alterations in human cancer. Nat. Biotechnol. 2012; 30:413–421. 22544022 10.1038/nbt.2203 PMC4383288

[30] Aran D., Sirota M., Butte A.J. Systematic pan-cancer analysis of tumour purity. Nat. Commun. 2015; 6:8971. 26634437 10.1038/ncomms9971 PMC4671203

[31] Adalsteinsson V.A., Ha G., Freeman S.S., Choudhury A.D., Stover D.G., Parsons H.A., Gydush G., Reed S.C., Rotem D., Rhoades J. et al. Scalable whole-exome sequencing of cell-free DNA reveals high concordance with metastatic tumors. Nat. Commun. 2017; 8:1324. 29109393 10.1038/s41467-017-00965-y PMC5673918

[32] Zhang H., Cisse M., Dauphin Y.N., Lopez-Paz D. mixup: beyond empirical risk minimization. 2017; arXiv doi:25 October 2017, preprint: not peer reviewed https://arxiv.org/abs/1710.09412.

[33] Menden K., Marouf M., Oller S., Dalmia A., Magruder D.S., Kloiber K., Heutink P., Bonn S. Deep learning-based cell composition analysis from tissue expression profiles. Sci. Adv. 2020; 6:eaba2619. 32832661 10.1126/sciadv.aba2619 PMC7439569

[34] Kanai Y., Arai E. Multilayer-omics analyses of human cancers: exploration of biomarkers and drug targets based on the activities of the International Human Epigenome Consortium. Front. Genet. 2014; 5:24. 24592273 10.3389/fgene.2014.00024 PMC3924033

[35] Bradford S.T., Nair S.S., Statham A.L., van Dijk S.J., Peters T.J., Anwar F., French H.J., von Martels J.Z.H., Sutcliffe B., Maddugoda M.P. et al. Methylome and transcriptome maps of human visceral and subcutaneous adipocytes reveal key epigenetic differences at developmental genes. Sci. Rep. 2019; 9:9511. 31266983 10.1038/s41598-019-45777-w PMC6606599

[36] Do C., Dumont E.L.P., Salas M., Castano A., Mujahed H., Maldonado L., Singh A., DaSilva-Arnold S.C., Bhagat G., Lehman S. et al. Allele-specific DNA methylation is increased in cancers and its dense mapping in normal plus neoplastic cells increases the yield of disease-associated regulatory SNPs. Genome Biol. 2020; 21:153. 32594908 10.1186/s13059-020-02059-3 PMC7322865

[37] Rizzardi L.F., Hickey P.F., Rodriguez DiBlasi V., Tryggvadóttir R., Callahan C.M., Idrizi A., Hansen K.D., Feinberg A.P. Neuronal brain-region-specific DNA methylation and chromatin accessibility are associated with neuropsychiatric trait heritability. Nat. Neurosci. 2019; 22:307–316. 30643296 10.1038/s41593-018-0297-8 PMC6348048

[38] Vaillancourt K., Chen G.G., Fiori L., Maussion G., Yerko V., Théroux J.-F., Ernst C., Labonté B., Calipari E., Nestler E.J. et al. Methylation of the tyrosine hydroxylase gene is dysregulated by cocaine dependence in the human striatum. iScience. 2021; 24:103169. 34693223 10.1016/j.isci.2021.103169 PMC8517202

[39] Rocks D., Jaric I., Tesfa L., Greally J.M., Suzuki M., Kundakovic M. Cell type-specific chromatin accessibility analysis in the mouse and human brain. Epigenetics. 2022; 17:202–219. 33775205 10.1080/15592294.2021.1896983 PMC8865312

[40] Ewing A.D., Smits N., Sanchez-Luque F.J., Faivre J., Brennan P.M., Richardson S.R., Cheetham S.W., Faulkner G.J. Nanopore sequencing enables comprehensive transposable element epigenomic profiling. Mol. Cell. 2020; 80:915–928. 33186547 10.1016/j.molcel.2020.10.024

[41] Li L., Li F., Xia Y., Yang X., Lv Q., Fang F., Wang Q., Bu W., Wang Y., Zhang K. et al. UVB induces cutaneous squamous cell carcinoma progression by de novo ID4 methylation via methylation regulating enzymes. EBioMedicine. 2020; 57:102835. 32574963 10.1016/j.ebiom.2020.102835 PMC7317242

[42] Vandiver A.R., Irizarry R.A., Hansen K.D., Garza L.A., Runarsson A., Li X., Chien A.L., Wang T.S., Leung S.G., Kang S. et al. Age and sun exposure-related widespread genomic blocks of hypomethylation in nonmalignant skin. Genome Biol. 2015; 16:80. 25886480 10.1186/s13059-015-0644-y PMC4423110

[43] Cao W., Lee H., Wu W., Zaman A., McCorkle S., Yan M., Chen J., Xing Q., Sinnott-Armstrong N., Xu H. et al. Multi-faceted epigenetic dysregulation of gene expression promotes esophageal squamous cell carcinoma. Nat. Commun. 2020; 11:3675. 32699215 10.1038/s41467-020-17227-z PMC7376194

[44] Goeppert B., Stichel D., Toth R., Fritzsche S., Loeffler M.A., Schlitter A.M., Neumann O., Assenov Y., Vogel M.N., Mehrabi A. et al. Integrative analysis reveals early and distinct genetic and epigenetic changes in intraductal papillary and tubulopapillary cholangiocarcinogenesis. Gut. 2022; 71:391–401. 33468537 10.1136/gutjnl-2020-322983 PMC8762040

[45] Jusakul A., Cutcutache I., Yong C.H., Lim J.Q., Huang M.N., Padmanabhan N., Nellore V., Kongpetch S., Ng A.W.T., Ng L.M. et al. Whole-genome and epigenomic landscapes of etiologically distinct subtypes of cholangiocarcinoma. Cancer Discov. 2017; 7:1116–1135. 28667006 10.1158/2159-8290.CD-17-0368 PMC5628134

[46] Yim J.H., Choi A.H., Li A.X., Qin H., Chang S., Tong S.-W.T., Chu P., Kim B.-W., Schmolze D., Lew R. et al. Identification of tissue-specific DNA methylation signatures for thyroid nodule diagnostics. Clin. Cancer Res. 2019; 25:544–551. 30093451 10.1158/1078-0432.CCR-18-0841 PMC6335179

[47] Schroeder D.I., Blair J.D., Lott P., Yu H.O.K., Hong D., Crary F., Ashwood P., Walker C., Korf I., Robinson W.P. et al. The human placenta methylome. Proc. Natl. Acad. Sci. U.S.A. 2013; 110:6037–6042. 23530188 10.1073/pnas.1215145110 PMC3625261

[48] Bowden S.A., Stockwell P.A., Rodger E.J., Parry M.F., Eccles M.R., Stayner C., Chatterjee A. Extensive inter-cyst DNA methylation variation in autosomal dominant polycystic kidney disease revealed by genome scale sequencing. Front. Genet. 2020; 11:348. 32351541 10.3389/fgene.2020.00348 PMC7174623

[49] Fang Q., Zhang X., Nie Q., Hu J., Zhou S., Wang C. Improved urine DNA methylation panel for early bladder cancer detection. BMC Cancer. 2022; 22:237. 35241014 10.1186/s12885-022-09268-y PMC8895640

[50] Skvortsova K., Masle-Farquhar E., Luu P.-L., Song J.Z., Qu W., Zotenko E., Gould C.M., Du Q., Peters T.J., Colino-Sanguino Y. et al. DNA hypermethylation encroachment at CpG island borders in cancer is predisposed by H3K4 monomethylation patterns. Cancer Cell. 2019; 35:297–314. 30753827 10.1016/j.ccell.2019.01.004

[51] Lin I.-H., Chen D.-T., Chang Y.-F., Lee Y.-L., Su C.-H., Cheng C., Tsai Y.-C., Ng S.-C., Chen H.-T., Lee M.-C. et al. Hierarchical clustering of breast cancer methylomes revealed differentially methylated and expressed breast cancer genes. PLoS One. 2015; 10:e0118453. 25706888 10.1371/journal.pone.0118453 PMC4338251

[52] Dong X., Shi M., Lee M., Toro R., Gravina S., Han W., Yasuda S., Wang T., Zhang Z., Vijg J. et al. Global, integrated analysis of methylomes and transcriptomes from laser capture microdissected bronchial and alveolar cells in human lung. Epigenetics. 2018; 13:264–274. 29465290 10.1080/15592294.2018.1441650 PMC5997142

[53] Lam D., Luu P.-L., Song J.Z., Qu W., Risbridger G.P., Lawrence M.G., Lu J., Trau M., Korbie D., Clark S.J. et al. Comprehensive evaluation of targeted multiplex bisulphite PCR sequencing for validation of DNA methylation biomarker panels. Clin Epigenet. 2020; 12:90. 10.1186/s13148-020-00880-y PMC7310104 32571390

[54] Jung N., Dai B., Gentles A.J., Majeti R., Feinberg A.P. An LSC epigenetic signature is largely mutation independent and implicates the HOXA cluster in AML pathogenesis. Nat. Commun. 2015; 6:8489. 26444494 10.1038/ncomms9489 PMC4633733

[55] de Goede O.M., Razzaghian H.R., Price E.M., Jones M.J., Kobor M.S., Robinson W.P., Lavoie P.M. Nucleated red blood cells impact DNA methylation and expression analyses of cord blood hematopoietic cells. Clin. Epigenet. 2015; 7:95. 10.1186/s13148-015-0129-6 PMC4567832 26366232

[56] Reinius L.E., Acevedo N., Joerink M., Pershagen G., Dahlén S.-E., Greco D., Söderhäll C., Scheynius A., Kere J. Differential DNA methylation in purified human blood cells: implications for cell lineage and studies on disease susceptibility. PLoS One. 2012; 7:e41361. 22848472 10.1371/journal.pone.0041361 PMC3405143

[57] Chatterjee A., Stockwell P.A., Rodger E.J., Duncan E.J., Parry M.F., Weeks R.J., Morison I.M. Genome-wide DNA methylation map of human neutrophils reveals widespread inter-individual epigenetic variation. Sci. Rep. 2015; 5:17328. 26612583 10.1038/srep17328 PMC4661471

[58] Zhang X., Ulm A., Somineni H.K., Oh S., Weirauch M.T., Zhang H.-X., Chen X., Lehn M.A., Janssen E.M., Ji H. DNA methylation dynamics during ex vivo differentiation and maturation of human dendritic cells. Epigenetics Chromatin. 2014; 7:21. 25161698 10.1186/1756-8935-7-21 PMC4144987

[59] Pacis A., Tailleux L., Morin A.M., Lambourne J., MacIsaac J.L., Yotova V., Dumaine A., Danckaert A., Luca F., Grenier J.-C. et al. Bacterial infection remodels the DNA methylation landscape of human dendritic cells. Genome Res. 2015; 25:1801–1811. 26392366 10.1101/gr.192005.115 PMC4665002

[60] Zhang Y., Maksimovic J., Naselli G., Qian J., Chopin M., Blewitt M.E., Oshlack A., Harrison L.C. Genome-wide DNA methylation analysis identifies hypomethylated genes regulated by FOXP3 in human regulatory T cells. Blood. 2013; 122:2823–2836. 23974203 10.1182/blood-2013-02-481788 PMC3798997

[61] Zebley C.C., Abdelsamed H.A., Ghoneim H.E., Alli S., Brown C., Haydar D., Mi T., Harris T., McGargill M.A., Krenciute G. et al. Proinflammatory cytokines promote TET2-mediated DNA demethylation during CD8 T cell effector differentiation. Cell Rep. 2021; 37:109796. 34644568 10.1016/j.celrep.2021.109796 PMC8593824

[62] Abdelsamed H.A., Zebley C.C., Nguyen H., Rutishauser R.L., Fan Y., Ghoneim H.E., Crawford J.C., Alfei F., Alli S., Ribeiro S.P. et al. Beta cell-specific CD8+ T cells maintain stem cell memory-associated epigenetic programs during type 1 diabetes. Nat. Immunol. 2020; 21:578–587. 32231298 10.1038/s41590-020-0633-5 PMC7183435

[63] Abdelsamed H.A., Moustaki A., Fan Y., Dogra P., Ghoneim H.E., Zebley C.C., Triplett B.M., Sekaly R.-P., Youngblood B. Human memory CD8 T cell effector potential is epigenetically preserved during in vivo homeostasis. J. Exp. Med. 2017; 214:1593–1606. 28490440 10.1084/jem.20161760 PMC5461005

[64] Jansen C.S., Prokhnevska N., Master V.A., Sanda M.G., Carlisle J.W., Bilen M.A., Cardenas M., Wilkinson S., Lake R., Sowalsky A.G. et al. An intra-tumoral niche maintains and differentiates stem-like CD8 T cells. Nature. 2019; 576:465–470. 31827286 10.1038/s41586-019-1836-5 PMC7108171

[65] Zhu T., Liu J., Beck S., Pan S., Capper D., Lechner M., Thirlwell C., Breeze C.E., Teschendorff A.E. A pan-tissue DNA methylation atlas enables in silico decomposition of human tissue methylomes at cell-type resolution. Nat. Methods. 2022; 19:296–306. 35277705 10.1038/s41592-022-01412-7 PMC8916958

[66] Yuan E., Matusiak M., Sirinukunwattana K., Varma S., Kidziński Ł., West R. Self-organizing maps for cellular In silico staining and cell substate classification. Front. Immunol. 2021; 12:765923. 34777384 10.3389/fimmu.2021.765923 PMC8588845

[67] Khan S.A., Tavolari S., Brandi G. Cholangiocarcinoma: epidemiology and risk factors. Liver Int. 2019; 39:19–31. 30851228 10.1111/liv.14095

[68] Clark S.J., Smallwood S.A., Lee H.J., Krueger F., Reik W., Kelsey G. Genome-wide base-resolution mapping of DNA methylation in single cells using single-cell bisulfite sequencing (scBS-seq). Nat. Protoc. 2017; 12:534–547. 28182018 10.1038/nprot.2016.187

[69] Smallwood S.A., Lee H.J., Angermueller C., Krueger F., Saadeh H., Peat J., Andrews S.R., Stegle O., Reik W., Kelsey G. Single-cell genome-wide bisulfite sequencing for assessing epigenetic heterogeneity. Nat. Methods. 2014; 11:817–820. 25042786 10.1038/nmeth.3035 PMC4117646

[70] Luo C., Keown C.L., Kurihara L., Zhou J., He Y., Li J., Castanon R., Lucero J., Nery J.R., Sandoval J.P. et al. Single-cell methylomes identify neuronal subtypes and regulatory elements in mammalian cortex. Science. 2017; 357:600–604. 28798132 10.1126/science.aan3351 PMC5570439

[71] Luo C., Rivkin A., Zhou J., Sandoval J.P., Kurihara L., Lucero J., Castanon R., Nery J.R., Pinto-Duarte A., Bui B. et al. Robust single-cell DNA methylome profiling with snmC-seq2. Nat. Commun. 2018; 9:3824. 30237449 10.1038/s41467-018-06355-2 PMC6147798

[72] Cisneros-Villanueva M., Hidalgo-Pérez L., Rios-Romero M., Cedro-Tanda A., Ruiz-Villavicencio C.A., Page K., Hastings R., Fernandez-Garcia D., Allsopp R., Fonseca-Montaño M.A. et al. Cell-free DNA analysis in current cancer clinical trials: a review. Br. J. Cancer. 2022; 126:391–400. 35027672 10.1038/s41416-021-01696-0 PMC8810765

[73] Egami S., Kawazoe H., Hashimoto H., Uozumi R., Arami T., Sakiyama N., Ohe Y., Nakada H., Aomori T., Ikemura S. et al. Peripheral blood biomarkers predict immune-related adverse events in non-small cell lung cancer patients treated with pembrolizumab: a multicenter retrospective study. J. Cancer. 2021; 12:2105–2112. 33754009 10.7150/jca.53242 PMC7974524

[74] Dart S.J., Cook A.M., Millward M.J., McDonnell A.M., Chin W.L., Hakeem M.U., Meniawy T.M., Bowyer S.E. Changes in expression of PD-L1 on peripheral T cells in patients with melanoma and lung cancer treated with PD-1 inhibitors. Sci. Rep. 2021; 11:15312. 34321489 10.1038/s41598-021-93479-z PMC8319434

[75] Sia D., Hoshida Y., Villanueva A., Roayaie S., Ferrer J., Tabak B., Peix J., Sole M., Tovar V., Alsinet C. et al. Integrative molecular analysis of intrahepatic cholangiocarcinoma reveals 2 classes that have different outcomes. Gastroenterology. 2013; 144:829–840. 23295441 10.1053/j.gastro.2013.01.001 PMC3624083

