# Supplementary material for: Neural-net-based cell deconvolution from DNA methylation reveals tumor microenvironment associated with cancer prognosis
Source: NAR Cancer. 2024 May 15;6(2):zcae022. doi: 10.1093/narcan/zcae022 (PMC11094754; doi:10.1093/narcan/zcae022)

Neural-net-based cell deconvolution from DNA methylation reveals tumor microenvironment associated with cancer prognosis

Yoshiaki Yasumizu[1,2,†], Masaki Hagiwara[1,3,4,†], Yuto Umezu[5], Hiroaki Fuji[6,7], Keiko Iwaisako[7,8], Masataka Asagiri[9], Shinji Uemoto[10], Yamami Nakamura[1], Sophia Thul[1], Azumi Ueyama[4,11], Kazunori Yokoi[12], Atsushi Tanemura[12], Yohei Nose[13], Takuro Saito[13], Hisashi Wada[11,13], Mamoru Kakuda[14], Masaharu Kohara[15], Satoshi Nojima[15], Eiichi Morii[15], Yuichiro Doki[13], Shimon Sakaguchi[1,16], Naganari Ohkura[1,3,*]

1. Department of Experimental Immunology, Immunology Frontier Research Center, Osaka University, Suita, Osaka, Japan
2. Integrated Frontier Research for Medical Science Division, Institute for Open and Transdisciplinary Research Initiatives (OTRI), Osaka University, Suita, Osaka, Japan
3. Department of Basic Research in Tumor Immunology, Graduate School of Medicine, Osaka University, Osaka, Japan
4. Pharmaceutical Research Division, Shionogi & Co., Ltd., Toyonaka, Osaka, Japan
5. Faculty of Medicine, Osaka University, Suita, Osaka, Japan
6. Department of Hepato-Biliary-Pancreatic Surgery, Hyogo Medical University, Nishinomiya, Hyogo, Japan
7. Division of Hepato-Biliary-Pancreatic Surgery and Transplantation, Department of Surgery, Graduate School of Medicine, Kyoto University, Kyoto, Kyoto, Japan
8. Faculty of Life and Medical Sciences, Doshisha University, Kyotanabe, Kyoto, Japan
9. Department of Pharmacology, Yamaguchi University Graduate School of Medicine, Ube, Yamaguchi, Japan
10. Shiga University Medical Science, Otsu, Shiga, Japan
11. Department of Clinical Research in Tumor Immunology, Graduate School of Medicine, Osaka University, Suita, Osaka, Japan
12. Department of Dermatology, Graduate School of Medicine, Osaka University, Suita, Osaka, Japan
13. Department of Gastroenterological Surgery, Graduate School of Medicine, Osaka University, Suita, Osaka, Japan
14. Department of Obstetrics and Gynecology, Graduate School of Medicine, Osaka University, Suita, Osaka, Japan
15. Department of Pathology, Graduate School of Medicine, Osaka University, Suita, Osaka, Japan
16. Department of Experimental Immunology, Institute for Life and Medical Sciences, Kyoto University, Kyoto, Kyoto, Japan

† These two authors contributed equally to this work

* Correspondence

TABLES

Table S1. Comprehensive Sample List of DNA Methylation Data

Samples used as a MEnet reference were listed.

Table S2. Source References for DNA Methylation Datasets

The original research articles of the DNA methylation dataset were listed.

Table S3. Clinical Profile of ICC Patients

Clinical information of ICC patients was shown.

SUPPLEMENTARY FIGURES

Figure S1. Comparative analysis of machine learning methods for predictive performance and computational efficiency

The predictive performance of various machine learning methods for purified cells (**A**) and mixed cells (**B**). Shown are mean squared error (MSE) outcomes and the corresponding test times for machine learning methods: Random Forest (RF), Support Vector Regression (SVR), Gradient Boosting (GB), Non-Negative Least Squares (NNLS), Neural Network without mixup (Neuralnet_wo_mixup), and Neural Network with mixup (NeuralNet_w_mixup), against purified or mixed samples. (**C**) The training times (seconds) for various machine learning methods (RF, SVR, GB, NNLS, Neuralnet_wo_mixup, and NeuralNet_w_mixup).

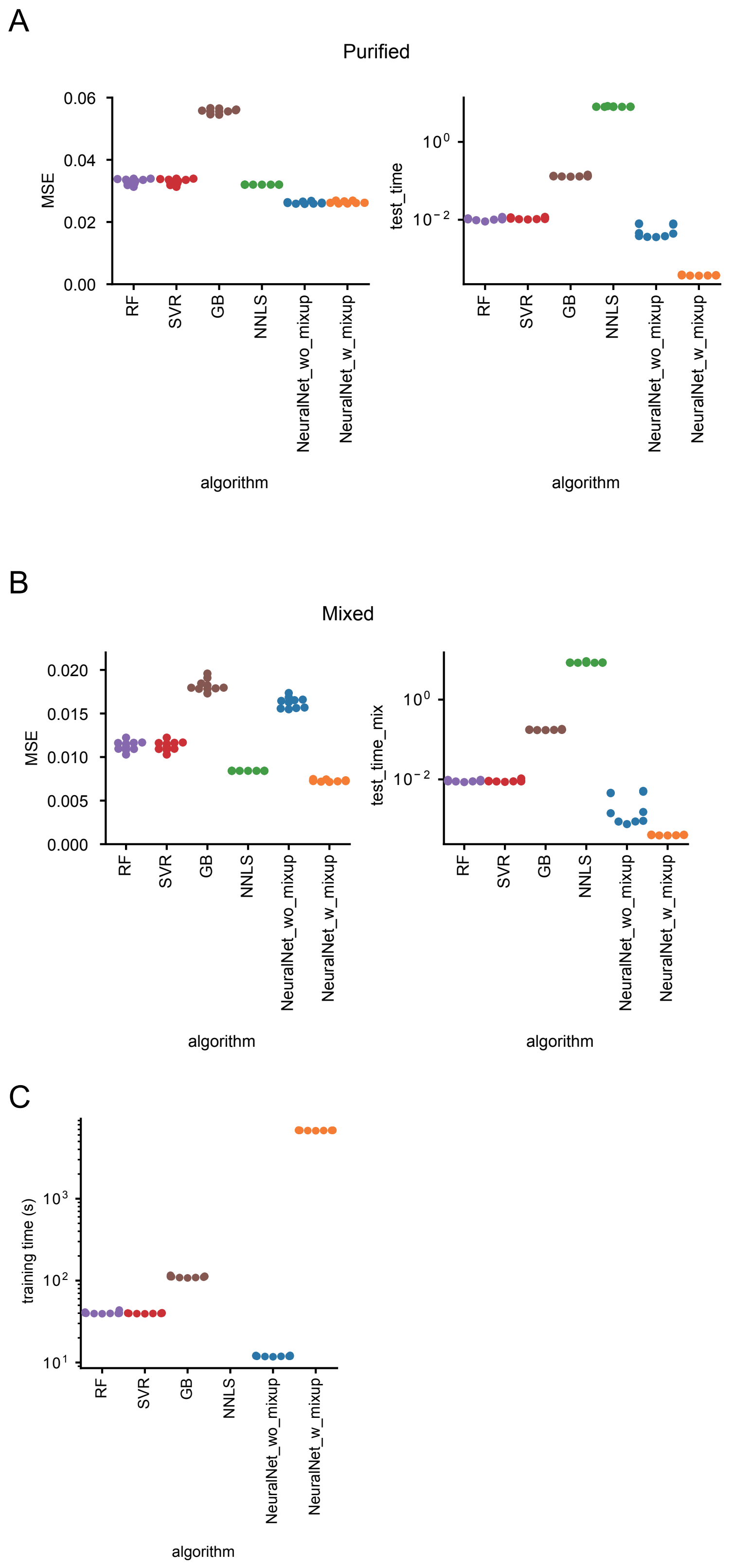

Figure S2. Performance evaluation of the neural network with mixup vs. established deconvolution methods in cell type prediction.

(**A**) Heatmap comparing the MSE of the Neural Network with mixup architecture with established deconvolution methods: ARIC, Constrained projection/quadratic programming (CP/QP), CIBERSORT, and RPC, for the prediction of purified (upper) and mixed cell samples (lower). (**B**) The panels show the MSE for distinguishing similar cell populations, such as CD4+ T cells, CD8+ T cells, B cells, and NK cells (upper), as well as Colon, Esophagus, Small Intestine, and Stomach cells (lower), in mixed samples. The performance of MEnet is compared with that of other methods. in mixed samples.

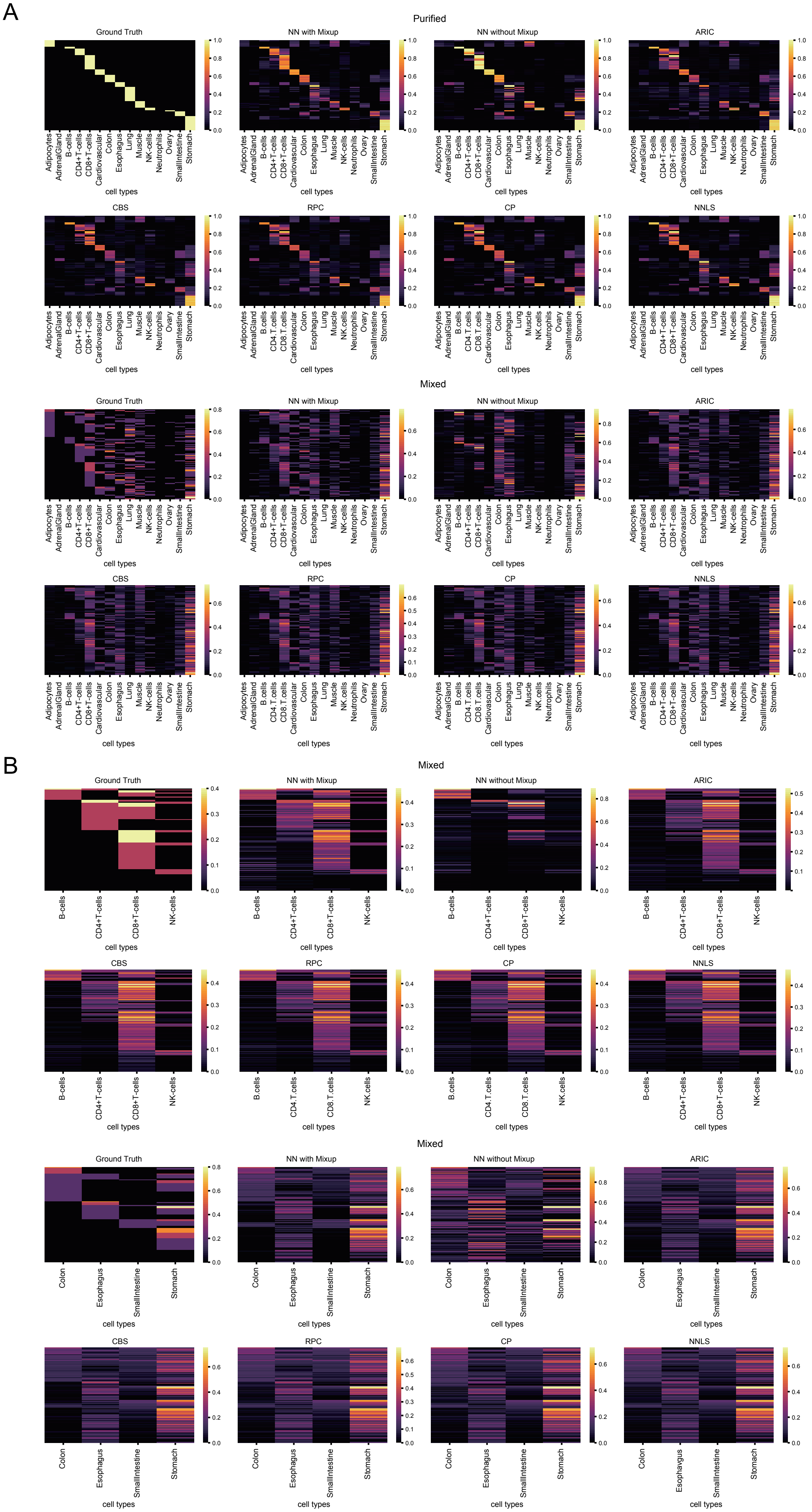
A
Purified
Ground Truth
NN with Mixup
NN without Mixup
ARIC
CBS
RPC
CP
NNLS
Mixed
Adipocytes
AdrenalGland
B-cells
CD4+T-cells
CD8+T-cells
Cardiovascular
Colon
Esophagus
Lung
Muscle
NK-cells
Neutrophils
Ovary
SmallIntestine
Stomach
cell types
B
Mixed
B-cells
CD4+T-cells
CD8+T-cells
NK-cells
Colon
Esophagus
SmallIntestine
Stomach

Figure S3. DNA methylation patterns of the major groups and genomic distribution in the feature regions of MEnet

(**A**) UMAP plot of the major group samples based on the DNA methylation rate in the feature regions. The plot displays the key cell populations identified in the dataset, with cell labels based on their cell surface markers or origins. (**B**) Bar plot showing the distribution of genomic partitions across the 87,726 feature regions identified from the whole genome, computed in 1,000 bp bins. These regions were chosen for their distinctiveness in various cell types, with up to 2,000 regions selected per cell type.

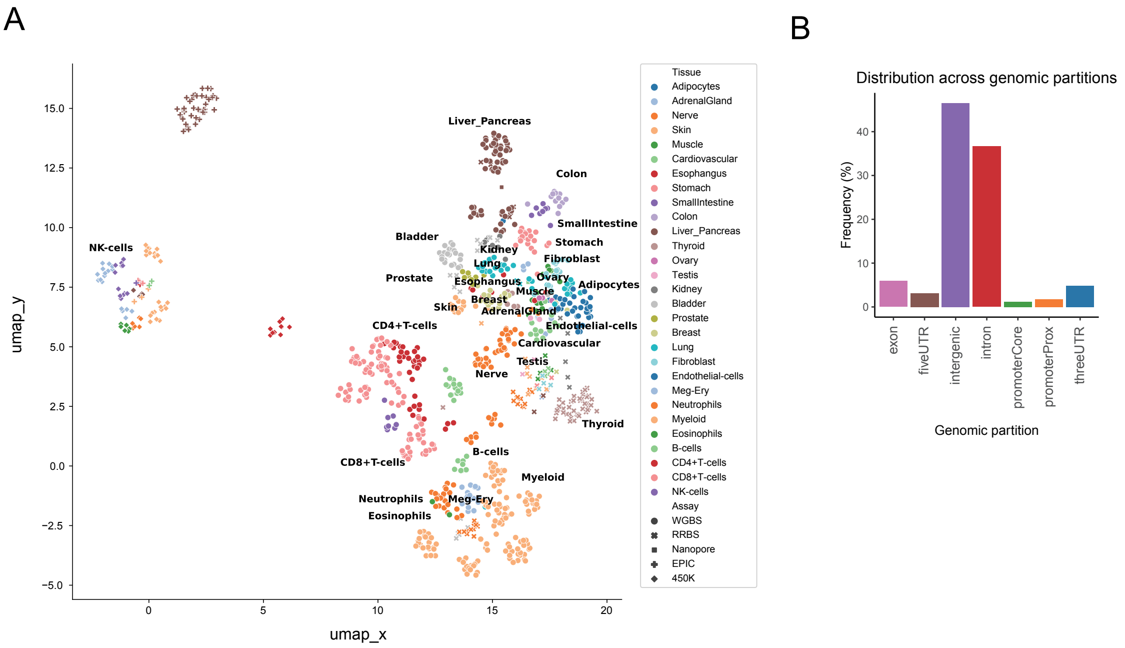

Figure S4. Details of Hyperparameter Tuning by Bayesian Optimization using Optuna.

(**A**) Shown are the transitions of the cross entropy between the predicted probability distribution and the distribution of the correct labels, referred to as OneHotCrossEntropy, for each parameter combination. (**B**) Bar plot representing the importance of each parameter to OneHotCrossEntropy. (**C**) Transition of OneHotCrossEntropy for each trial.

A

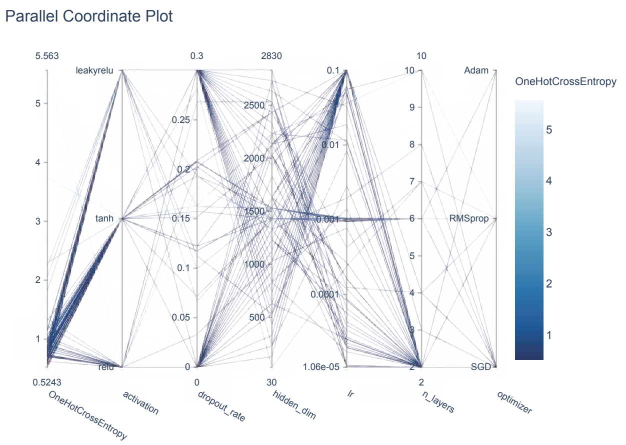

B

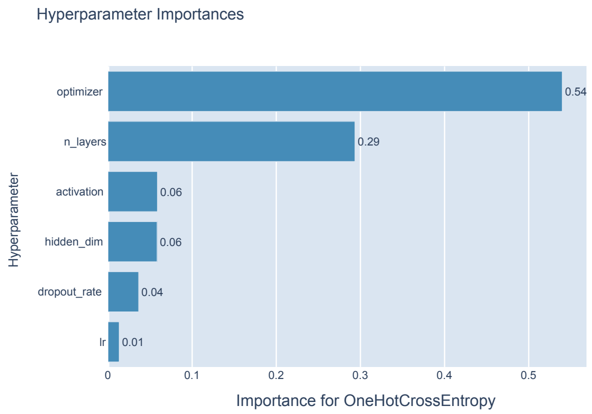

C

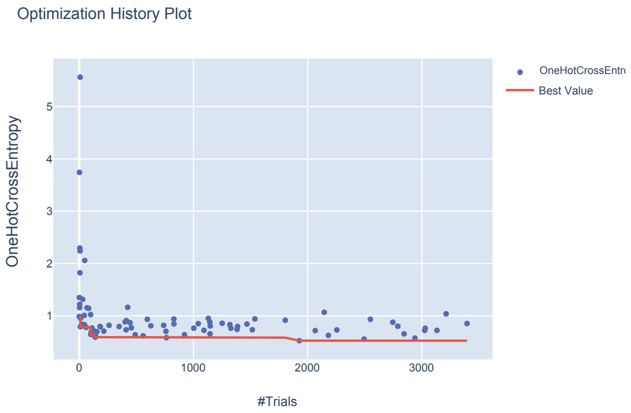

Figure S5. Subsampling analysis for determining the required read depth for the prediction using various methods.

Root mean squared error (RMSE) of the prediction for subsampled reads from whole genome bisulfite sequencing (WGBS) and reduced representation bisulfite sequencing (RRBS) samples not used in model training, using (**A**) ARIC, (**B**) CIBERSORT, (**C**) RPC, and (**D**) CP/QP methods. For each method, the top row represents the results of subsamples using purified cell data, while the bottom row depicts the results of simulated mixture data. The left panel in each row shows the results at the Major Group level and the right panel at the Minor Group level. Various proportions of sequence reads are sampled with five independent seeds and evaluated for accuracy. The dotted line across the panels represents the average RMSE for 500 random predictions. Markers represent independent samples.

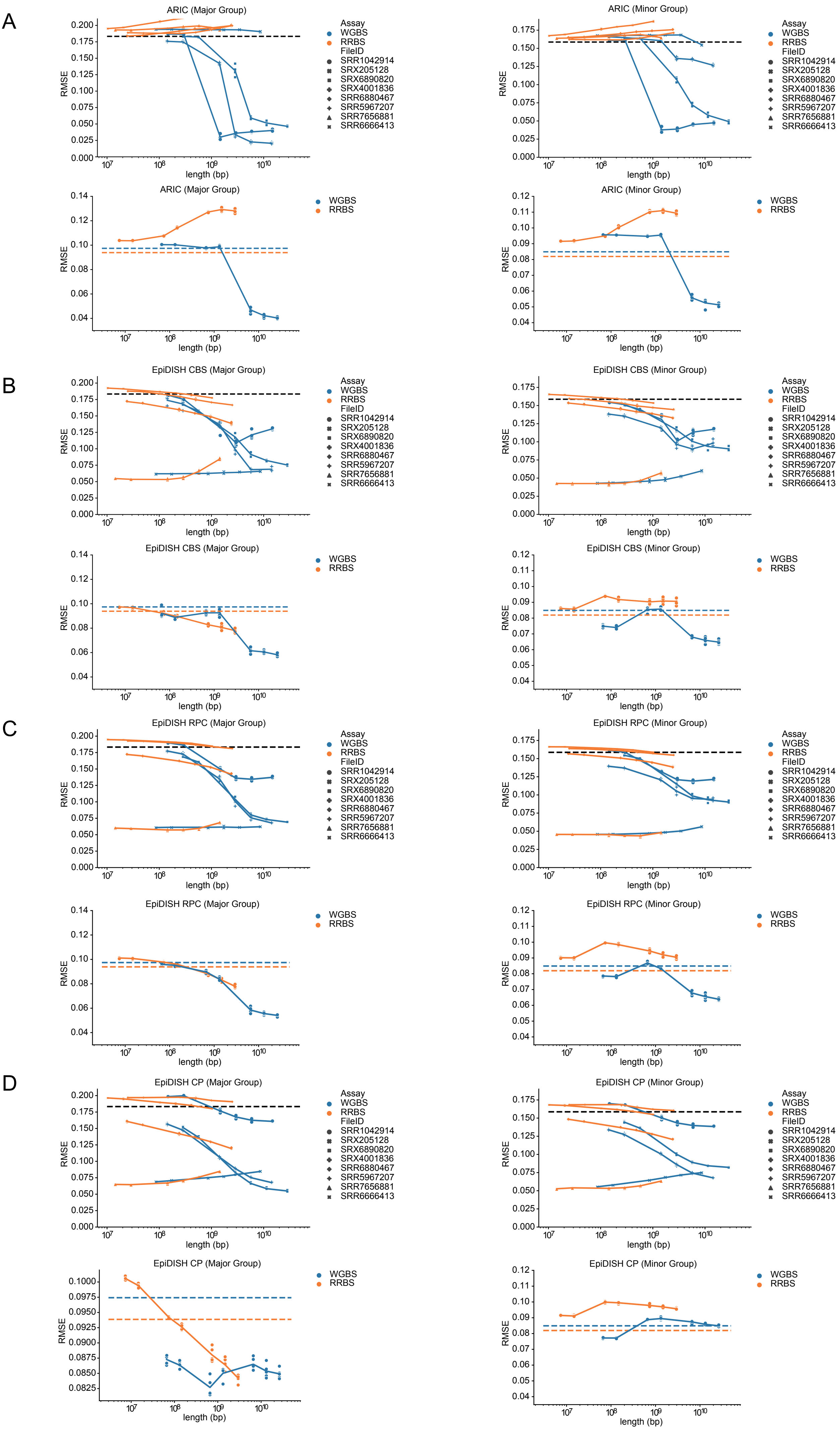

A
ARIC (Major Group)
ARIC (Minor Group)
RMSE
length (bp)
Assay
WGBS
RRBS
FileID
SRR1042914
SRX205128
SRX6890820
SRX4001836
SRR6880467
SRR5967207
SRR7656881
SRR6666413
B
EpiDISH CBS (Major Group)
EpiDISH CBS (Minor Group)
C
EpiDISH RPC (Major Group)
EpiDISH RPC (Minor Group)
D
EpiDISH CP (Major Group)
EpiDISH CP (Minor Group)

Figure S6. Estimation and validation of immune cell proportions by MEnet.

(**A**) Scatter plot showing the proportion of artificially mixed immune cells (y-axis) and the proportion of immune cells estimated from DNA methylation (x-axis). This data was generated using sorted immune cells from PBMCs (n=12). (**B**) Scatter plots showing the proportion of tumor-infiltrated immune cells measured by flow cytometry (y-axis) and the proportion of immune cells estimated from DNA methylation (x-axis). n=11. (**C**) Bar plot showing the deconvolution results for each sorted circulating CD4+ T cell subset. DNA was directly sequenced with a nanopore sequencer. Circulating CD4+ T cell subsets: CD4+ CD25high CD45RA- (activated Treg), CD4+ CD25- CD45RA- (memory Tconv), and CD4+ CD25- CD45RA+ (naive Tconv).

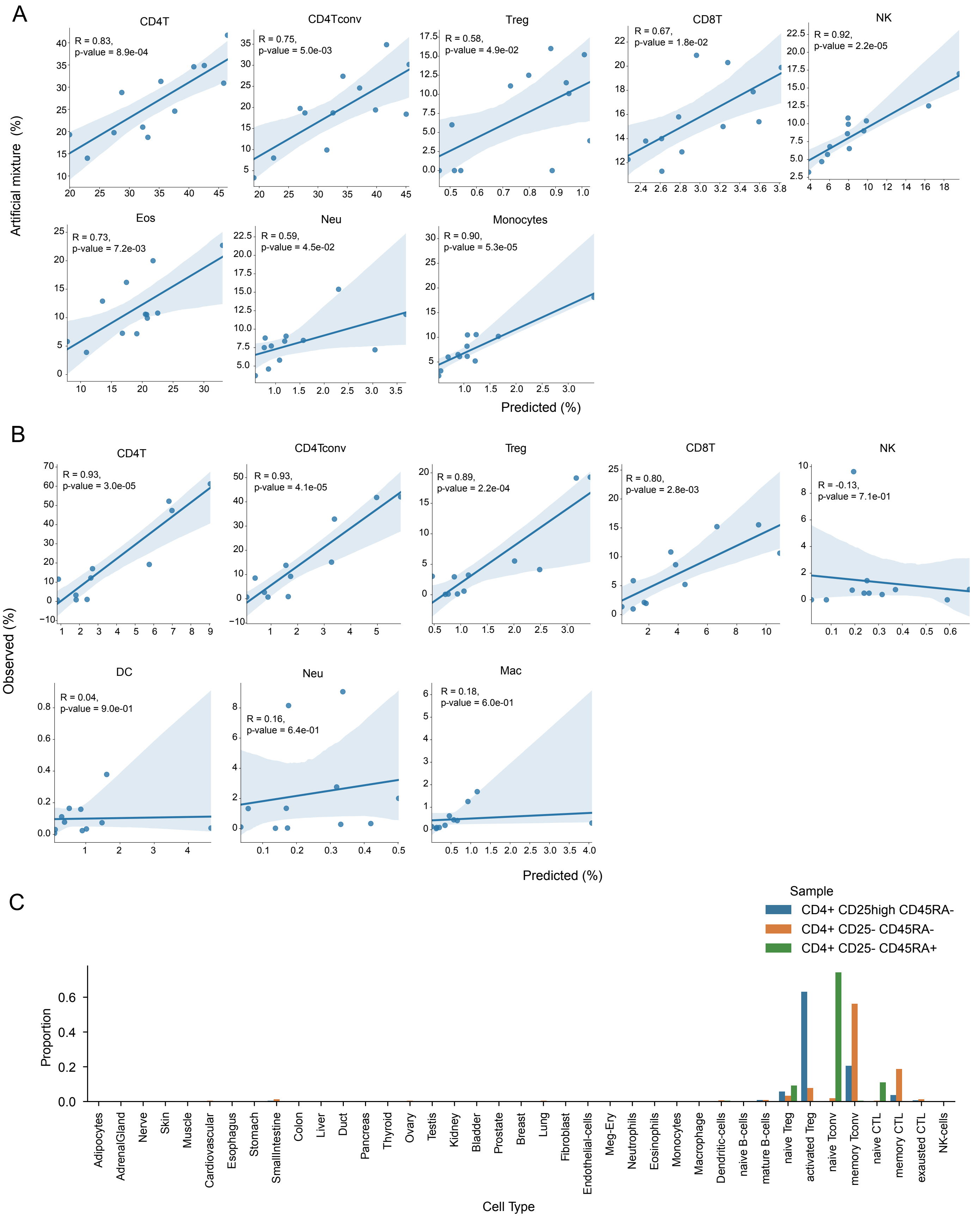

Figure S7. Correlation of the predicted cell frequencies with clinical features

(**A**) Heatmap showing Spearman' s correlation coefficients between clinically derived metrics and predicted cell frequencies obtained by MEnet. The examined clinical metrics include tumor stage, tumor diameter, blood markers for liver function, and tumor markers such as carcinoembryonic antigen (CEA) and CA19-9.

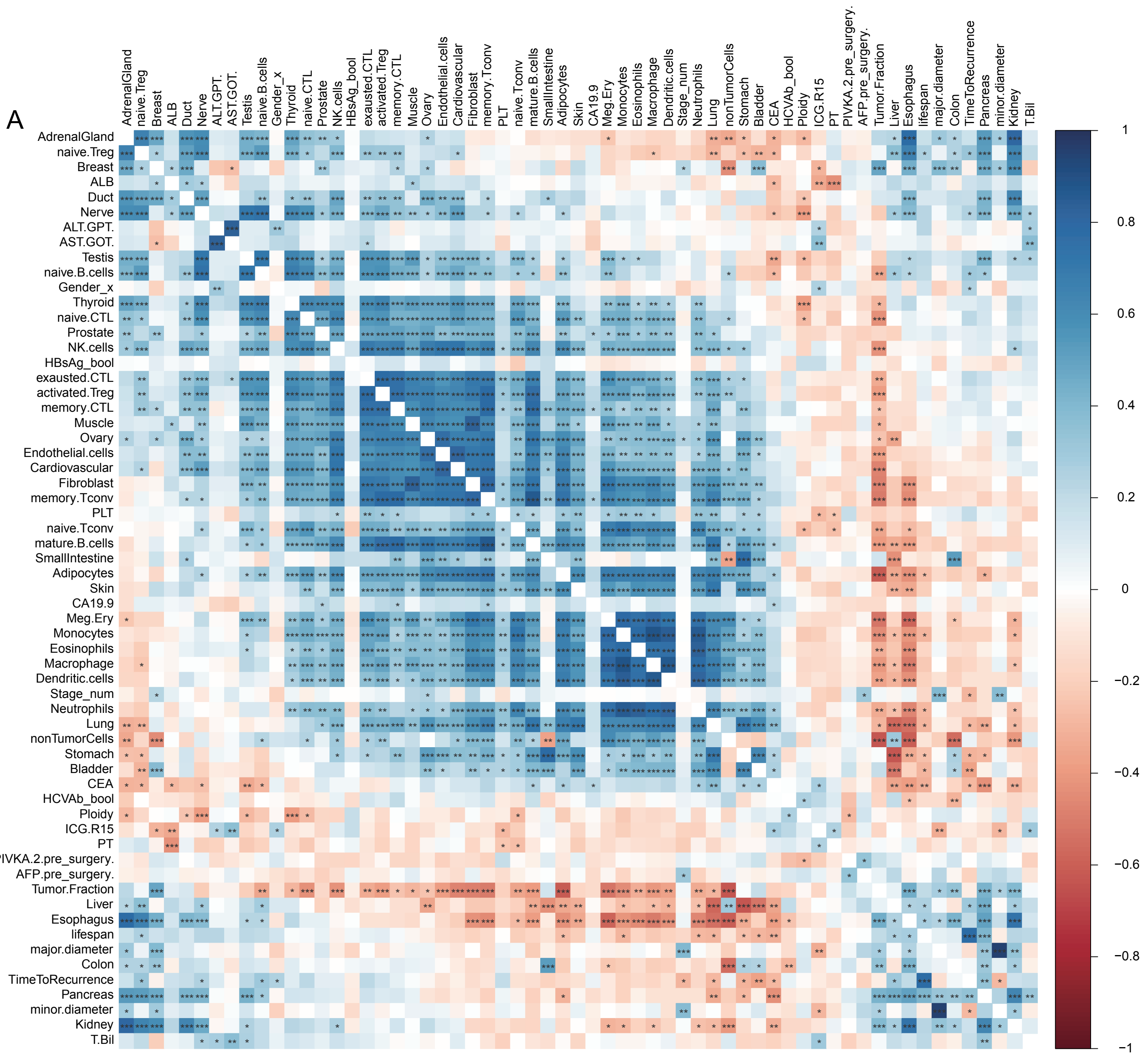

Figure S8. Cox-hazard analysis and concordance-index distribution for recurrence prediction based on the cell proportion

(**A**) Forest plot of the Cox-Hazard analysis for recurrence duration. The analysis incorporates NMFs and cancer stages (quantified as integers from 1 to 4) as covariates. (**B**) Distribution of the Concordance-index (C-index) for recurrence prediction, calculated by bootstrap. Bootstrap involved performing Cox hazard regression on 62 randomly selected samples and calculating the C-index on 10 independent samples, repeated 100 times. The distribution of the C-index calculated from MEnet results and cancer stage was tested using the Mann-Whitney U-test.

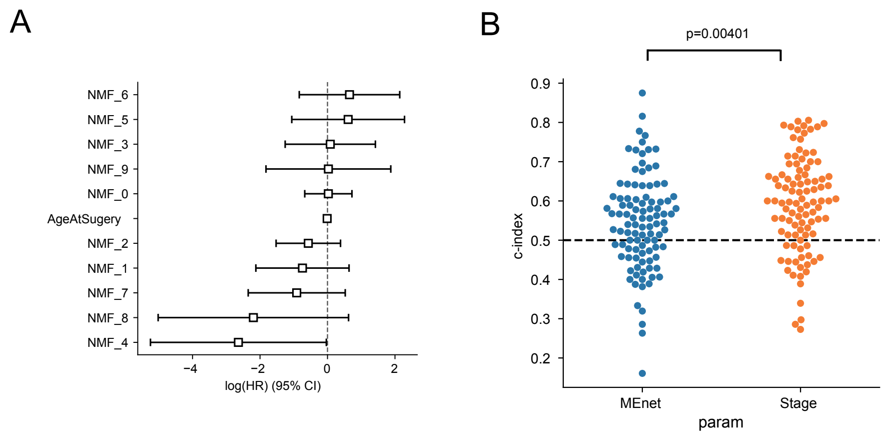

Supplement: zcae022_Supplemental_Files [file zcae022_supplemental_files.zip › 240418_figs_sup-comp_v2.pdf]
